# Recovery of genomes from metagenomes via a dereplication, aggregation and scoring strategy

**DOI:** 10.1038/s41564-018-0171-1

**Published:** 2018-05-28

**Authors:** Christian M. K. Sieber, Alexander J. Probst, Allison Sharrar, Brian C. Thomas, Matthias Hess, Susannah G. Tringe, Jillian F. Banfield

**Affiliations:** 1 Department of Energy, Joint Genome Institute, Walnut Creek, CA USA; 2 Department of Earth and Planetary Science, University of California, Berkeley, CA USA; 3 Department of Animal Science, University of California, Davis, CA USA

**Keywords:** Genome assembly algorithms, Bioinformatics, Software, Metagenomics, Environmental microbiology

## Abstract

Microbial communities are critical to ecosystem function. A key objective of metagenomic studies is to analyse organism-specific metabolic pathways and reconstruct community interaction networks. This requires accurate assignment of assembled genome fragments to genomes. Existing binning methods often fail to reconstruct a reasonable number of genomes and report many bins of low quality and completeness. Furthermore, the performance of existing algorithms varies between samples and biotopes. Here, we present a dereplication, aggregation and scoring strategy, DAS Tool, that combines the strengths of a flexible set of established binning algorithms. DAS Tool applied to a constructed community generated more accurate bins than any automated method. Indeed, when applied to environmental and host-associated samples of different complexity, DAS Tool recovered substantially more near-complete genomes, including previously unreported lineages, than any single binning method alone. The ability to reconstruct many near-complete genomes from metagenomics data will greatly advance genome-centric analyses of ecosystems.

## Abstract (web summary)

Here the authors present a tool that enables a flexible set of existing binning algorithms to be combined, resulting in improved binning accuracy and the recovery of more near-complete genomes from metagenomes compared to standalone methods.

## Main

Genome-resolved metagenomics targets the reconstruction of genomes from environmental shotgun DNA sequence data. Based on the genome sequence, metabolic pathways of individual organisms can be inferred and their lifestyle in the microbial community can be predicted. The challenge of recovering genomes from complex mixtures of sequence fragments is comparable to that of assembling jigsaw puzzles from a mixture of many puzzles without knowing how many puzzles are present and what they look like. Not surprisingly, powerful bioinformatics methods are required to achieve the desired outcome.

Early approaches primarily made use of shared GC content and coverage^1^, but binning contigs from more complex ecosystems required advanced methods taking sequence composition such as tetranucleotide frequencies into account^2,3^. Sequence compositional analysis was implemented within emergent self-organizing maps (ESOMs) to successfully extract genomes from metagenomes^4^. The ESOM-based approach, involving user-defined clustering, has been widely used to recover draft genomes from many different environments but has limitations for high-complexity data sets such as from soil or sediments^5,6^. A major advance in binning methods came with the realization that the pattern of organism abundances across a sample series was a binning signature^7,8^.

Phylogenetic profile information was of minimal use early in the metagenomics era because the number of reference microbial genomes was very small. However, the phylogenetic signal continues to grow in utility as the number of reference genome sequences increases.

Current state-of-the-art binners combine sequence abundance and composition into one model^9–12^, and some of them additionally use marker genes from a reference database^13,14^. The quality assessment in terms of completeness and contamination of predicted bins is essential and can be estimated based on the frequency of single-copy marker genes^15,16^.

Existing binning tools are based on broadly accepted features and clustering algorithms, and benchmarked using data sets analysed in their respective publications. In fact, most binning methods have been demonstrated using relatively simple communities (for example, premature infant gut data sets^7^). However, the value of bins generated when these methods are applied to other samples is uncertain. Here, we tested the performance of a set of well-established binning methods by applying them to data from a group of ecosystems that varied dramatically in complexity. We found that no single approach performed well on all ecosystems. Furthermore, many incomplete bins and multi-genome mega bins were predicted. The different binning performance and the fact that different tools reconstruct different genomes with varying levels of completeness motivated the development of a strategy that integrates the results of predictions of multiple binning algorithms.

Probst et al. combined and curated the results of three binning methods and increased the total number of reconstructed near-complete genomes from a subsurface aquifer environment over that obtained by using just one method^17^. An automated binning combination approach was able to reduce the overall contamination in bins but also decreased the overall completeness^18^. These findings motivated the development of the dereplication, aggregation and scoring tool (DAS Tool). DAS Tool is an automated method that integrates a flexible number of binning algorithms to calculate an optimized, non-redundant set of bins from a single assembly. We show that this approach generates a larger number of high-quality genomes than achieved using any single tool.

## Results

### Development of an integrative binning approach

**Fig. 1:**
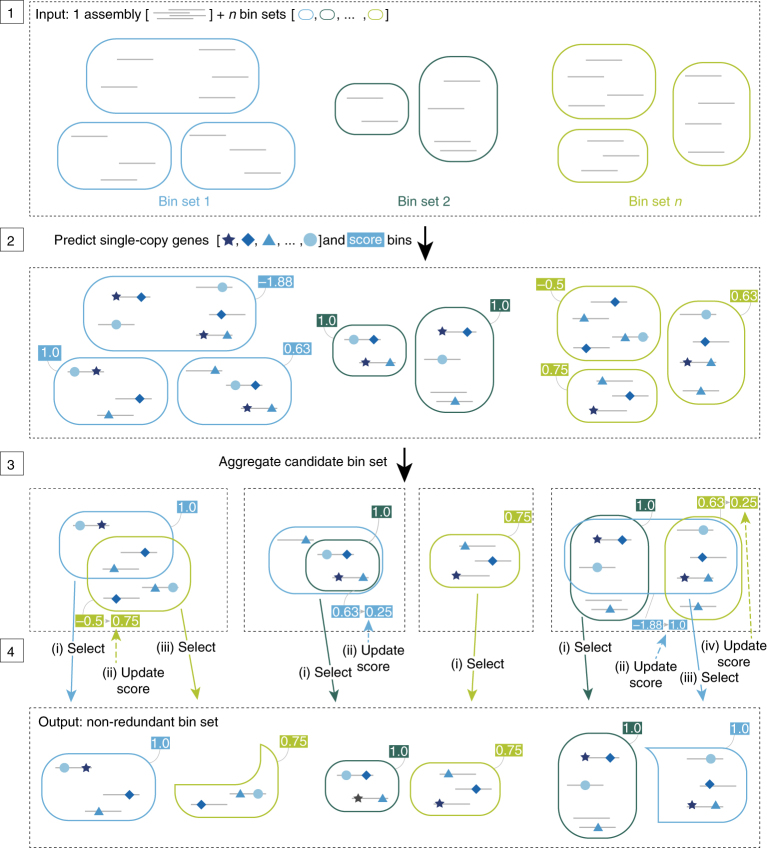
Overview of the DAS Tool algorithm. Step 1: The input of the DAS Tool comprises scaffolds of one assembly (grey lines) and a variable number of bin sets from different binning predictions (same-coloured rounded rectangles). Step 2: Single-copy genes (blue shapes) on scaffolds are predicted and scores (blue and green boxes) are assigned to bins. Step 3: Aggregation of redundant candidate bin set from all binning predictions. Step 4: Iterative selection of high-scoring bins and updating of scores of remaining partial candidate bins. The output comprises non-redundant set of high-scoring bins from different input predictions.

The DAS Tool approach to solve the binning problem is to integrate predictions from multiple established binning tools. The number and type of binning tools is flexible. Candidate bins are generated independently when all binning tools are applied to the same assembly. DAS Tool then uses a consensus approach to select a single set of non-redundant, high-quality bins (Fig. 1). Nevertheless, we advise that the user examine each of the final bins to identify potential contamination based on erroneous phylogenetic affiliation and to remove sequences from phage/virus (based on gene content).

### DAS Tool applied to simulated microbial communities

**Fig. 2:**
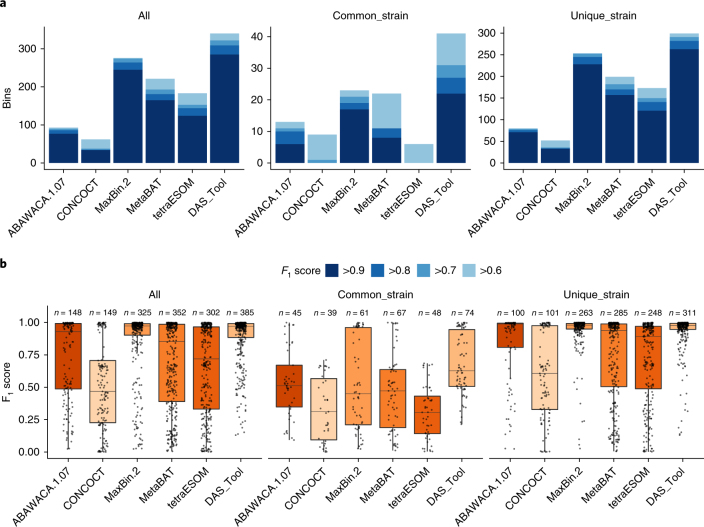
Reconstructed genomes from a simulated microbial community consisting of 596 genomes. **a**, The number of reconstructed genomes per method above a certain F_1_ score threshold. The higher the F_1_ score the more similar the reconstructed genome is to the reference. **b**, The distribution of F_1_ scores of all reported bins (centre line, median; box limits, upper and lower quartiles; whiskers, 1.5× interquartile range). Individual values appear as dots. The precise *n* number in terms of reconstructed bins per method is given above each boxplot. Metrics are calculated for all reference genomes (all), genomes with strain variation (common_strain; ≤95% average nucleotide identity (ANI) to other reference genomes) and without strain variation (unique_strain; >95% ANI to other reference genomes).

To validate the DAS Tool algorithm, we applied it to three assemblies from simulated microbial communities that were created for the CAMI challenge^19^. The assemblies comprise different numbers of organisms including strain variation to simulate microbial communities with low (40 genomes), medium (132 genomes) and high complexity (596 genomes). We predicted bins using five binning tools (ABAWACA 1.07 (https://github.com/CK7/abawaca), CONCOCT^9^, MaxBin 2^13^, MetaBAT^10^ and tetranucleotide ESOMs^4^) and combined the result using DAS Tool. To determine how well the reconstructed bins represent the reference genomes, we calculated F_1_ scores, which are the harmonic mean of precision and recall. We also focused on how well each tool reconstructs genomes with common or unique strains in the data set. For the most challenging, high-complexity data set, DAS Tool reports more high-quality genomes with and without strain variation than any individual tool (Fig. 2). DAS Tool reports 41 high-quality bins (F_1_ score > 0.6) of genomes with common strains and 299 genomes of unique strains. MaxBin 2 obtained the second-best results with 23 and 253 genomes (F_1_ score > 0.6) for reference genomes with common and unique strains, respectively. Tetranucleotide ESOMs performed well in reconstructing genomes from unique strains (173 genomes, F_1_ score > 0.6), but reported only a low number of the genomes with strain variation (6 genomes, F_1_ score > 0.6) (Fig. 2). Besides reconstructing a higher number of high-quality genomes, the F_1_ score distribution of all reconstructed genomes shows an equal or higher median compared to the best-performing single binning tool (DAS Tool: 0.627 (common strain), 0.979 (unique strain); MaxBin 2: 0.449 (common strain), 0.980 (unique strain)) (Fig. 2). DAS Tool not only reconstructs a higher number of high-quality genomes and resolves strain variation better than any of the individual tools on the high-complexity data set, but also performs better on the assemblies of medium- and low-complexity communities (Supplementary Fig. 1).

### Application of DAS Tool to environmental metagenomic data

Probst et al.^17^ generated a highly curated set of genome bins from metagenomic data from a high-CO_2_ cold-water geyser that were ideal for evaluation of the DAS Tool algorithm. The data comprise two assemblies of sequences from samples collected sequentially on 3.0 μm and 0.2 μm filters and a set of 3.0 μm filtrates from subsurface fluids collected at a single time point. The published bins were generated by a comparative approach of three methods followed by manual curation of the results^17^. We used CheckM^15^ to generate marker gene-based quality estimates for the published bins that can be compared to quality estimates for all binning methods, including DAS Tool. Bins were only considered to be of high (>90% complete) or draft (70–90% complete) quality if they had less than 5% contamination.

**Fig. 3:**
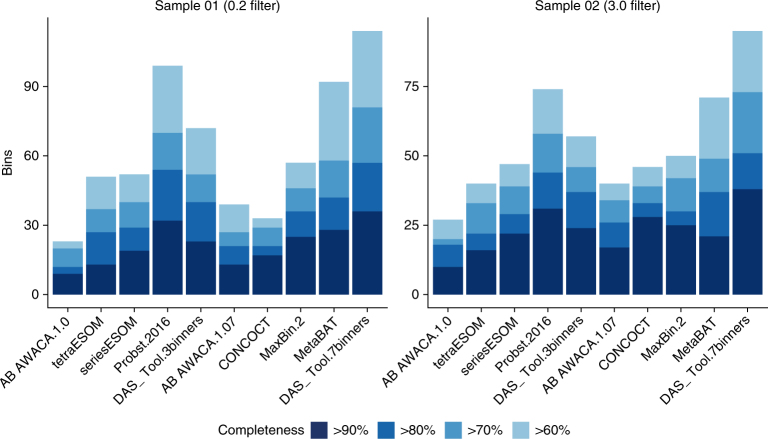
Reconstructed genomes from Crystal Geyser, a high-CO_2_ cold-water geyser. The number of high-quality genomes with low contamination (<5%) from metagenomic assemblies of two samples. Probst.2016 represents the combination from ref. ^17^ of ABAWACA.1, tetraESOM and seriesESOM and a final manual curation step. DAS_Tool.3binners uses the same three predictions as input. DAS_Tool.7binners additionally uses ABAWACA.2, CONCOCT, MaxBin.2 and MetaBat.

We compared the results of the three independent binning predictions from ref. ^17^ (ABAWACA 1.0, tetranucleotide ESOMs, differential-abundance ESOMs), as well as those from ABAWACA 1.07, CONCOCT, MetaBAT and MaxBin 2 to results achieved using DAS Tool. DAS Tool was applied using either a combination of three or seven different binning algorithms (Fig. 3 and Supplementary Table 2).

Although DAS Tool with three binning algorithms reported more near-complete and draft genomes than the three methods alone, it returned fewer genomes than in the curated set from ref. ^17^ (Fig. 3 and Supplementary Table 2). However, when we included seven binning tools in DAS Tool (adding ABAWACA 1.07, CONCOCT, MaxBin 2 and MetaBAT), the reported number of near-complete genomes was higher for the 0.2 μm sample (DAS Tool: 36 genomes, Probst: 32) and even higher for the 3.0 μm sample (DAS Tool: 38, Probst: 31). For both samples a larger number of draft genomes was reconstructed than was achieved previously^17^ (Fig. 3 and Supplementary Table 2). The number of draft genomes increased slightly when allowing more contamination per bin (Supplementary Fig. 3).

### Combination of bins using DAS Tool improves genome count from metagenomic data with different levels of complexity

To evaluate the performance of DAS Tool on samples of different complexity, we applied it to shotgun metagenomic data of lower, medium and high complexity from human microbiomes^20^, natural oil seeps^21,22^ and soil (see Data availability). We binned all samples separately using ABAWACA 1.07, CONCOCT, MaxBin 2, MetaBAT and tetranucleotide ESOMs. All predictions were combined using DAS Tool and CheckM was used to estimate the quality of the resulting bins. In addition, we used ggKbase binning tools to analyse the human gut data. This was appropriate, given colonization of the human gut by genomically well-characterized bacteria. ggKbase tools were not used in the other analyses because they do not perform well in systems with many previously unreported organisms.

**Fig. 4:**
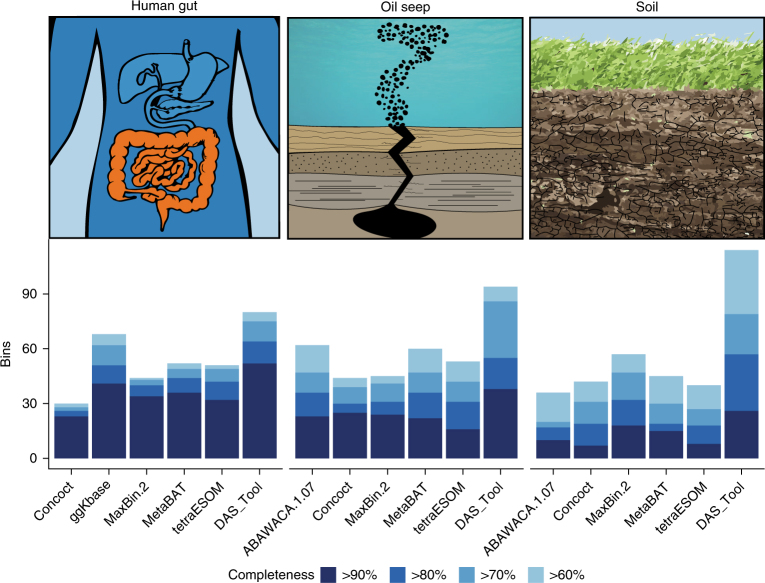
The number of high-quality genomes with low contamination (<5%) from metagenomic assemblies of samples from three ecosystems representing a range of complexity. Samples were collected from adult human gut (1 faecal sample), oil seeps (5 samples) and hillslope soil and underlying weathered shale (6 samples). The samples were assembled and binned separately. Reconstructed genomes were summed up per ecosystem. For sample-by-sample results, see Supplementary Fig. 5.

Summing up the number of bins of each quality level that were generated for the three ecosystems, DAS Tool reported the highest number of near-complete and draft bins in all cases (Fig. 4).

Interestingly, the performance of the single binning tools that were used as input for DAS Tool differed between ecosystems and none of them was the clear winner. This is also reflected in the composition of the final bin set in terms of the input methods where genomes were selected (Supplementary Fig. 4). In the case of bins generated for the lower-complexity human gut samples using single binning tools, ggKbase followed by MetaBAT generated the largest number of near-complete genomes. For the medium-complexity oil seeps, ABAWACA 1.07 and MetaBAT produced the most draft-quality genomes while CONCOCT produced slightly more high-quality bins. For high-complexity soil data, MaxBin 2 reported the most draft and near-complete genomes.

We also examined the performance of the various binning approaches sample by sample. DAS Tool reported either the most or the same number of near-complete genomes with low contamination for all 12 samples (higher: 6/12; equal: 6/12). The number of reconstructed genomes per sample increases when considering genomes with a higher amount of contamination. In 11 of 12 samples, DAS Tool reports a higher number of genomes with more than 70% completeness and less than 15% contamination (Supplementary Fig. 6).

To estimate the expected species number per ecosystem we clustered for each assembly all predicted ribosomal protein S3 sequences at 99% amino acid identity. Given the number of resulting clusters and the number of draft genomes, DAS Tool reconstructed 76.5% (75 bins/98 clusters), 24.6% (86/349) and 8.7% (79/907) of possible genomes from the data sets of human gut, oil seeps and soil, respectively (Supplementary Table 3).

Besides CheckM, we also estimated the completeness of bins using the single-copy gene base approach BUSCO^16^. In general, the estimations of BUSCO are less conservative, which results in a higher number of classified high-quality genomes compared to CheckM. According to BUSCO, DAS Tool reports the most near-complete and draft-quality genomes for all ecosystems (Supplementary Fig. 7a).

We also applied the recently published Binning_refiner^18^ to combine the binning results of the three environments and compared its performance to DAS Tool. For all 12 assemblies, DAS Tool extracted considerably more near-complete and draft genomes than Binning_refiner (Supplementary Fig. 8).

### Genome analysis reveals previously unreported lineage with hydrocarbon degradation potential

Binning of metagenomic data from Santa Barbara oil seep samples revealed three genomes whose 16S rRNA gene sequences lacked closely related sequences in the SILVA database^23^ (78.8, 79.4 and 87.4% identity). The estimated completeness of these reconstructed genomes ranges from 95.6 to 89.6% (Supplementary Table 4).

In a phylogenetic tree based on 16 concatenated ribosomal proteins, the three genomes cluster as a monophyletic group with one TA06 and two WOR-3 genomes (Supplementary Fig. 9a). The JGI_Cruoil_03_Bacteria_38_101 forms a cluster together with the TA06 lineage at a pairwise tree distance (patristic distance) of 1.2977 but is more distant to the two WOR-3 (patristic distances of 1.5531 and 1.5258, respectively). In contrast, the two lineages JGI_Cruoil_03_Bacteria_44_89 and JGI_Cruoil_03_Bacteria_51_56 share greater similarity with the two WOR-3 at a minimal patristic distance of 1.3350 and 1.0582, respectively, and have a greater distance to the TA06 (patristic distance of 1.4328 and 1.4673, respectively).

For comparison, the patristic distance between representatives of closely related phyla in the same tree was between 1.0282 and 1.2110 (Firmicute *Thermincola* sp. JR versus the Chloroflexus *C. aurantiacus* J-10-fl and Melainabacteria *Obscuribacter phosphatis* versus the Cyanobacteria *Leptolyngbya* sp. PCC 7104) (Supplementary Fig. 10).

Given that both distances are smaller than the distances of TA06 and WOR-3 to our reconstructed genomes JGI_Cruoil_03_Bacteria_38_101 and JGI_Cruoil_03_Bacteria_44_89 as well as the distance of JGI_Cruoil_03_Bacteria_38_101 to JGI_Cruoil_03_Bacteria_44_89 (patristic distance of 1.5164), we conclude that these two genomes may be representatives of two previously unreported phylum-level lineages. The third genome, JGI_Cruoil_03_Bacteria_51_56, is closer to the WOR-3 at a patristic distance of 1.0582 and is probably part of the WOR-3 candidate division.

Interestingly, the 16S rRNA gene sequences of all three of our reconstructed genomes group with some sequences classified as TA06 and one sequence classified as a WS3 (the other WS3 sequences form a lineage sibling to Zixibacteria) (Supplementary Figs. 9b and 11). Except for one TA06 (Candidate_division_TA06_bacterium_32_111), the corresponding TA06 and WS3 genomes place distant from our genomes on the concatenated ribosomal protein tree. Thus, some of the 16S rRNA gene sequences of these publicly available genomes may be misclassified or misbinned (a common problem with 16S rRNA gene binning, especially if the gene is in multi-copy and the scaffolds are short). Regardless, it is clear that our genomes are highly distinct from any other genomes in public databases.

Pathway analysis reveals genes encoding for hydrocarbon degradation enzymes, including aldehyde dehydrogenase, which are present in all three genomes. Additionally, alcohol dehydrogenase, aldehyde ferredoxin oxidoreductase and methanol dehydrogenase are present in JGI_Cruoil_03_Bacteria_44_89, the genome with highest estimated completeness, suggesting pathways for degradation of alkanes and methanol (Supplementary Table 5).

### Genomes from soil

From six soil samples, we reconstructed 79 minimally contaminated (<5%) draft genomes (>70% completeness), 26 of which were high-quality draft genomes (>90% completeness) (Supplementary Fig. 5). Two of the high-quality genomes were well-assembled (a Gemmatimonadetes genome consisting of 11 scaffolds and a Bacteroidetes genome on 14 scaffolds), with estimated completeness above 97% and contamination below 3.3%.

It has been shown recently that some Gemmatimonadetes are able to consume methanol using a pyrrolo-quinoline quinone (PQQ)-dependent methanol dehydrogenase (MDH) and to convert the resulting formaldehyde using the tetrahydromethanopterin (THMPT) and tetrahydrofolate (THF)-linked formaldehyde oxidation pathways^24^. Likewise, we were able to find a PQQ-MDH and two key enzymes of the THF pathway (methenyltetrahydrofolate cyclohydrolase, methylenetetrahydrofolate dehydrogenase) in the high-quality Gemmatimonadetes genome bin but could not find any enzymes belonging to the THMPT pathway. Additionally, we found genes for carbon fixation, fermentation, nitrogen assimilation, complex carbon degradation and sulfur metabolism. Similarly, the Bacteroidetes genome encodes enzymes for carbon fixation, fermentation and nitrogen assimilation, but by contrast has no genes for methane metabolism, complex carbon degradation or sulfur metabolism (Supplementary Table 5).

## Discussion

We tested a group of currently available, published metagenomics binning algorithms to evaluate how well they performed when applied to samples of a wide range of complexity. Consistent with previous work showing that use of differential coverage signals can significantly improve binning outcomes^7,8^, the single binning algorithms that used these signals (CONCOCT, MaxBin, MetaBAT, ABAWACA) performed better than composition-based tools (tetra-ESOM) on most samples. However, it is notable that each of these was variably effective across the different system types, and even among different samples from the same ecosystem, and no single binning algorithm was consistently the most effective. Therefore, we do not suggest an optimal set of binning methods for use as input for DAS Tool. However, because of the overall solid performance of MaxBin in our study and in the recently published CAMI challenge^19^, MaxBin combined with two or three other binning methods may serve as a solid basis for DAS Tool. Interestingly, for the simple human gut community that includes organisms that are closely related to genomically characterized species, the manual combination of phylogeny, GC, coverage and single-copy gene inventory produces very good binning outcomes; however, this is not the case for more complex data sets.

DAS Tool, the consensus binning strategy presented here, almost always extracted considerably more genomes from complex metagenomes than any of the single binning tools alone. While DAS Tool did not outperform manual bin combination and curation when using the same starting set of bins from three single binning approaches, adding four additional binning algorithms resulted in more near-complete bins than the published manually curated results. This finding underlines the advantage of including more binning methods in DAS Tool. It is important to note that even tools that generate only a small number of high-quality bins can significantly improve the results of DAS Tool because other tools sometimes miss these bins.

It is not uncommon for the research community to question the quality of genomes reconstructed from metagenomes. Imperfect bins are a challenge for all studies that attempt to genomically resolve complex ecosystems. However, if they can be obtained, the value of high-quality draft genomes is enormous. Different single algorithm methods not only generate different numbers of bins, but the genome content can differ slightly. This variable performance can be evaluated by using strategies such as DAS Tool. In picking the best bins from each binning tool, DAS Tool is able to equalize performance variations of single binning tools and thus increase the total number of near-complete genomes recovered. Because it uses a single-copy gene-based scoring function it is able to distinguish between high- and low-quality bins and by using an appropriate score cutoff it can filter out low-quality bins and control the number of megabins.

Despite improvements in assembling and binning methods, reconstructing genomes from soil metagenomics data is still challenging. With the help of DAS Tool we were able to extract dozens of high-quality genomes from soil, including some near-complete genomes. Furthermore, in re-analysing public data from off-shore oil seep sediments we identified and genomically characterized organisms of a previously unreported lineage that is probably involved in hydrocarbon degradation.

In conclusion, DAS Tool can integrate manual binning methods such as ESOMs and can incorporate the results of any contig-based binning algorithm. Thus, it is highly scalable and can make use of binning tools developed in the future.

## Methods

### Implementation

DAS Tool is implemented in R (ref. ^25^). Besides R-base functions, we used the R-packages doMC^26^ to implement multicore functionality, data.table^27^ for efficient data access and storage and ggplot2^28^ to visualize results. DAS Tool is available from https://github.com/cmks/DAS_Tool.

### Scoring function

To estimate the quality and completeness of predicted bins we set up a single-copy gene (SCG) based scoring function (equation (1)). The idea behind the scoring function is to rank genome bins based on their estimated completeness and contamination. Therefore, the bin score increases with the number of SCGs but decreases with the number of duplicate SCGs per bin:

1 $${S}_{{\rm{b}}}=\frac{{\rm{uSCG}}}{{\rm{rSCG}}}-b\frac{{\rm{dSCG}}}{{\rm{uSCG}}}-c\frac{{\rm{\Sigma }}{\rm{SCG}}-{\rm{uSCG}}}{{\rm{rSCG}}}$$

The function calculates a bin score based on the frequency of 51 bacterial or 38 archaeal reference single-copy genes (rSCG). The first term of the function represents the fraction of SCGs present and accounts for the completeness of the genome. It is the number of unique single-copy genes per bin (uSCG) divided by the number of reference SCGs (rSCG). The second term accounts for contamination and decreases the score in the case of duplicated SCGs (dSCG). This is calculated as the ratio of the number of duplicated SCGs (dSCG) divided by the total number of unique SCGs (uSCG) in a bin. The third term is a penalty for megabins and is the total number of extra single-copy genes divided by the number of reference genes. It is calculated as the difference of the total number of predicted SCGs (ΣSCG) and the number of unique SCGs per bin divided by the number of reference SCGs. Both penalty terms are accompanied by weighting factors (*b*,*c*). For each bin, scores using the bacterial and archaeal reference gene set are calculated and the greater of the two scores is reported as the bin score.

### Marker gene prediction

Genes in the assembly are predicted using prodigal^29^ with the meta option and the ‘-m’ flag for preventing gene models to be built over ambiguous nucleotides. SCGs are determined using databases of bacterial^30^ and archaeal SCGs^17^ as a seed to select candidates of SCGs from the metagenomes using USEARCH^31^ (e-value 1e-2). The candidates were then searched^31^ against the entire database (e-value 1e-5) and called present if the query spanned at least 50% of the alignment with the best hit in the database.

Although all results shown in this manuscript are based on USEARCH^31^, DAS Tool can also make use of the open-source tools DIAMOND^32^ and BLAST^33^ to predict SCGs. Scripts for SCG prediction are available from https://github.com/AJProbst/sngl_cp_gn.

### Selection algorithm

In the first step, a redundant candidate bin set is created, which consists of all predicted bins of the input binning methods. The quality of all bins in the candidate set is estimated using the SCG-based scoring function (equation (1)).

An iterative procedure is then used to select a non-redundant bin set (Fig. 1). The highest scoring bin is first extracted out of the candidate set. If two or more bins have the same score, the bin with a higher scaffold N50 value is chosen. The N50 value is the minimum contig length needed to cover 50% of the genome bin size with contigs equal or larger than this value. If the N50 value is also equal, the larger bin in terms of nucleotide sequence is selected. After removing the bin from the set, all contigs that belong to this bin are also removed from other bins. Because this step influences the composition of other bins, the scoring function is applied again on all altered bins. The iteration continues as long as selected bins are above a score of zero or until all bins in the candidate set are selected. During the iteration process, bins above a predefined score threshold *t* are selected into the final bin set.

### Parameter estimation

To determine the optimal values for weighting factors *b* and *c*, and the score threshold *t*, we performed a grid search over a range of parameters. We applied DAS Tool with the range of parameters (*b*, *c* ∈ {0, 0.1,…,3}, *t* ∈ {0, 0.1,…,0.9}) on data from a synthetic microbial community that was constructed by mixing together DNA of 22 bacteria (including different species from the same genus) and 3 archaea^34^ and evaluated the quality of the selected bins. Higher values of *b* and *c* resulted in higher average precision and recall of reconstructed bins (Supplementary Fig. 12a–d), but a lower total number of high-quality bins (Supplementary Fig. 12e,f). In contrast, a higher score threshold leads to higher average precision and recall of bins but lower number of total reported high-quality bins (Supplementary Fig. 12). We selected parameters that maximize the sum of the fraction of reconstructed high-quality bins, precision and recall. In general, the performance of DAS Tool was very robust to parameter variations on this relatively small data set of 25 genomes. Therefore, no unique optimum but a range of parameters (*b*, *c* ∈ {0.4,0.5,0.6}, *t* ∈ {0.3,0.4,0.5,0.6}) could be determined that maximize bin number, precision and recall. The analyses in this study were performed using *b* = 0.5, *c* = 0.5 and *t* = 0.5.

### Assembly and mapping

The reads of the synthetic community and soil samples were quality filtered by SICKLE (version 1.21, https://github.com/najoshi/sickle, default parameters) and assembled using IBDA_UD^35^. All samples were assembled separately. Read mapping for all samples was done using Bowtie 2^36^.

### Binning

To generate input bin sets for DAS Tool we applied the automated binning tools ABAWACA 1.07 (https://github.com/CK7/abawaca), CONCOCT^9^ (version 0.4.0), MaxBin 2^13^ (version 2.1.1) and MetaBAT^10^ (version 0.25.4). The automated binning tools are based on different clustering algorithms and features. ABAWACA performs a hierarchical clustering on tetranucleotide frequencies and differential coverage, and takes marker genes into account. CONCOCT uses Gaussian mixture models and tetranucleotides frequencies with differential coverage^9^. MaxBin 2 is based on an expectation-maximization algorithm and uses tetranucleotides, differential coverage and marker genes^13^. MetaBAT applies a k-medoid clustering on tetranucleotide frequencies and differential coverage^10^. We also calculated tetranucleotide ESOMs^4^ and selected clusters manually using Databionic ESOM Tools^37^. Additionally, we manually binned the human gut microbiome data based on GC, coverage and taxonomic profile using ggKbase tools^38^ (http://ggkbase.berkeley.edu). All binning tools were run using default parameters. ABAWACA 1.07 returned no results on the human gut data due to the lack of differential coverage information. The bins of ABAWACA 1.0, tetranucleotide ESOMs and differential-abundance ESOMs for the Crystal Geyser data were obtained from ref. ^17^. For comparison purposes we also combined bins of the human gut, oil seep and soil assemblies using Binning_refiner^18^. Because Binning_refiner can only combine up to three binning predictions at once, we first combined the bins of CONCOCT, MetaBAT and tetranucleotide ESOMs and combined that result with MaxBin 2 and ABAWACA 1.07.

### Binning evaluation

We used three simulated metagenomic data sets consisting of 40, 132 and 596 genomes of the CAMI (Critical Assessment of Metagenome Interpretation) challenge^19^. We downloaded the gold standard assemblies and the assignment of assembled contigs to reference genomes from data.cami-challenge.org and used this information to calculate the accuracy of reconstructed bins.

For each bin *B*_b_ of the set of predicted bins *B*, we determined the highest fraction in terms of nucleotides that belong to a certain genome *G*_g_ from the set of reference genomes *G*. Based on the sequence lengths of *B*_b_ and *G*_g_ we calculated the *F*_1_ score (equation (2)), which is the harmonic mean of precision (equation (3)) and recall (equation (4)).

2 $${F}_{1}\,{{\rm{Score}}}_{{\rm{b}}}=2\frac{{P}_{{\rm{b}}}{R}_{{\rm{b}}}}{{P}_{{\rm{b}}}+{R}_{{\rm{b}}}}$$

3 $${P}_{{\rm{b}}}=\frac{{\rm{length}}\left({B}_{{\rm{b}}}\cap {G}_{{\rm{g}}}\right)}{{\rm{length}}\left({B}_{{\rm{b}}}\right)},\,{\rm{where}}\,g={{\rm{argmax}}}_{i\in G}\left(\frac{{\rm{length}}\left({G}_{i}\cap {B}_{{\rm{b}}}\right)}{{\rm{length}}\left({B}_{{\rm{b}}}\right)}\right)$$

4 $${R}_{{\rm{b}}}=\frac{{\rm{length}}\left({B}_{{\rm{b}}}\cap {G}_{{\rm{g}}}\right)}{{\rm{length}}\left({G}_{{\rm{b}}}\right)},\,{\rm{where}}\,g={{\rm{argmax}}}_{i\in G}\left(\frac{{\rm{length}}\left({G}_{i}\cap {B}_{{\rm{b}}}\right)}{{\rm{length}}\left({B}_{{\rm{b}}}\right)}\right)$$

Because DAS Tool only selects bacterial and archaeal genomes, all bins that map to circular elements were removed from the evaluation. To determine how well the binning tools resolve strain variation we not only calculated *F*_1_ scores on the entire set of reference genomes but also on subsets of genomes with and without common strains in the data set. The classification of reference genomes belonging to the set of unique strains (<95% average nucleotide identity (ANI) to other genomes) or common strains (≥95% ANI) was obtained from data.cami-challenge.org.

For real metagenomics data sets where the ground truth in terms of genome composition is unknown, we estimated genome completeness based on marker genes using the lineage workflow of CheckM^15^ and the Bacteria odb9 data set of BUSCO^16^. Completeness and contamination of BUSCO results was calculated based on the percentage of present and duplicate marker genes per bin.

### Estimation of species number per ecosystem

We estimated the expected species number per assembly in calculating operational taxonomic units (OTUs) based on ribosomal protein S3 (RPS3). We used the software rpS3_trckr (https://github.com/AJProbst/rpS3_trckr) to predict RPS3 sequences and cluster them at 99% amino acid identity for generating RPS3 based OTUs.

### Genome curation and annotation

Assemblies of submitted genomes were error-corrected using re_assemble_errors.py (https://github.com/christophertbrown/fix_assembly_errors). Gene prediction was performed with the same settings used for marker gene prediction in DAS Tool (prodigal^29^ in meta mode and ‘-p’ flag). Functional predictions were made using the ggKbase annotation pipeline, which uses USEARCH^31^ to search predicted open reading frames against Kegg^39^, UniRef100^40^ and UniProt^41^.

### Phylogenetic tree

The ribosomal protein tree is based on concatenated alignments of the amino acid sequences of 16 ribosomal proteins (ribosomal proteins L2, S3, L3, L4, L5, L6P-L9E, L15, L16-L10E, S8, L14, L18, L22, L24, S10, S19 and S17). Alignments were created for each protein using MUSCLE^42^ and trimmed manually. After concatenation, columns with more than 95% gaps were removed. We calculated the phylogenetic tree using the maximum likelihood algorithm RAxML^43^ on the CIPRES web server^44^ in choosing the LG (PROTCATLG) evolutionary model and autoMRE to automatically determine the number of bootstraps. 16S rRNA gene sequences were aligned using SSU-align^45^, trimmed and submitted to the CIPRES web server^44^. We used RAxML^43^ and the GTRGAMMA model and determined the number of bootstraps using autoMRE.

Pairwise distances in terms of the sum of branch lengths between two taxa in the phylogenetic tree (patristic distance) were calculated using the cophenetic.phylo function of the ape R-package^46^.

### Reporting Summary

Further information on experimental design is available in the Nature Research Reporting Summary linked to this article.

### Code availability

DAS Tool is available from https://github.com/cmks/DAS_Tool (version 1.1 was used in this analysis: https://github.com/cmks/DAS_Tool/releases/tag/1.1.0) and as Supplementary Code.

### Data availability

The reads of human gut samples (SRA accession no. SRR3496379)^20^ and Crystal geyser samples (BioProjects PRJNA229517 and PRJNA297582)^17^ and the synthetic community for parameter estimation (SRA accession no. SRX1836716)^34^ were obtained from NCBI. Reads of the oil seep data (Gold Analysis Project ID nos. Ga0004151, Ga0004152, Ga0004153, Ga0005105 and Ga0005106)^21,22^ and soil samples (Gold Analysis Project ID nos. Ga0007435, Ga0007436, Ga0007437, Ga0007438, Ga0007439 and Ga0007440) were downloaded from JGI portal pages (https://img.jgi.doe.gov/cgi-bin/m/main.cgi). Assemblies were downloaded from ggKbase for the human gut samples (http://ggkbase.berkeley.edu/LEY3/organisms) and from IMG for the oil seep samples (Gold Study ID no. Gs0090292). Genomes from oil seep and soil samples that were analysed in this study are available on ggKbase (http://ggkbase.berkeley.edu/dastool) and NCBI (GenBank accession nos. NGFL00000000, NOZP00000000, NOZQ00000000, NGFH00000000 and NGFI00000000).

## Supplementary information

Supplementary Information Supplementary Figures 1–9, Supplementary Figure 12, Supplementary Tables 3 and 4

Reporting Summary

Supplementary Figure 10 Full-size phylogenetic trees based on 16 concatenated ribosomal protein sequences

Supplementary Figure 11 Full-size phylogenetic trees based on based on 16S rRNA gene sequence

Supplementary Table 1 Accuracy of reconstructed genomes per method based on F1 score for simulated microbial communities of low, medium and high complexity

Supplementary Table 2 Number of high-quality genomes with low contamination (<5%) from metagenomic assemblies of samples from ecosystems representing a range of complexity. Samples were collected from a high CO_2_ cold water geyser (two samples), adult human gut (one faecal sample), oil seeps (five samples), and hillslope soil and underlying weathered shale (six samples). Samples were assembled and binned separately. ‘Probst.2016’ represents the combination by Probst et al. 2016 of ABAWACA.1, tetraESOM and seriesESOM and a final manual curation step. DAS_Tool.3binners uses the same three predictions as input. DAS_Tool.7binners additionally uses ABAWACA.1.07, CONCOCT, MaxBin.2 and MetaBat

Supplementary Table 5 Predicted key enzymes of metabolic pathways of five reconstructed genomes from oil-seep and soil metagenomes

Supplementary Code Source code of DAS Tool. For latest version please see https://github.com/cmks/DAS_Tool

## References

[1] Tyson GW (2004). Community structure and metabolism through reconstruction of microbial genomes from the environment. Nature.

[2] Teeling H, Meyerdierks A, Bauer M, Amann R, Glöckner FO (2004). Application of tetranucleotide frequencies for the assignment of genomic fragments. Environ. Microbiol..

[3] Abe T (2002). A novel bioinformatic strategy for unveiling hidden genome signatures of eukaryotes: self-organizing map of oligonucleotide frequency. Genome Inform..

[4] Dick GJ (2009). Community-wide analysis of microbial genome sequence signatures. Genome Biol..

[5] Anantharaman K, Breier JA, Dick GJ (2016). Metagenomic resolution of microbial functions in deep-sea hydrothermal plumes across the Eastern Lau Spreading Center. ISME J..

[6] Hug LA (2015). Critical biogeochemical functions in the subsurface are associated with bacteria from new phyla and little studied lineages. Env. Microbiol..

[7] Sharon I (2013). Time series community genomics analysis reveals rapid shifts in bacterial species, strains, and phage during infant gut colonization. Genome Res..

[8] Albertsen M (2013). Genome sequences of rare, uncultured bacteria obtained by differential coverage binning of multiple metagenomes. Nat. Biotechnol..

[9] Alneberg J (2014). Binning metagenomic contigs by coverage and composition. Nat. Methods.

[10] Kang DD, Froula J, Egan R, Wang Z (2015). MetaBAT, an efficient tool for accurately reconstructing single genomes from complex microbial communities. PeerJ.

[11] Lu YY, Chen T, Fuhrman JA, Sun F (2017). COCACOLA: binning metagenomic contigs using sequence COmposition, read CoverAge, CO-alignment and paired-end read LinkAge. Bioinformatics.

[12] Graham ED, Heidelberg JF, Tully BJ (2017). BinSanity: unsupervised clustering of environmental microbial assemblies using coverage and affinity propagation. PeerJ.

[13] Wu YWW, Simmons BA, Singer SW (2015). MaxBin 2.0: an automated binning algorithm to recover genomes from multiple metagenomic datasets. Bioinformatics.

[14] Lin HH, Liao YC (2016). Accurate binning of metagenomic contigs via automated clustering sequences using information of genomic signatures and marker genes. Sci. Rep..

[15] Parks DH, Imelfort M, Skennerton CT, Hugenholtz P, Tyson GW (2015). CheckM: assessing the quality of microbial genomes recovered from isolates, single cells, and metagenomes. Genome Res.

[16] Simao FA, Waterhouse RM, Ioannidis P, Kriventseva EV, Zdobnov EM (2015). BUSCO: assessing genome assembly and annotation completeness with single-copy orthologs. Bioinformatics.

[17] Probst AJ (2017). Genomic resolution of a cold subsurface aquifer community provides metabolic insights for novel microbes adapted to high CO_2_ concentrations. Environ. Microbiol..

[18] Song WZ, Thomas T (2017). Binning_refiner: improving genome bins through the combination of different binning programs. Bioinformatics.

[19] Sczyrba A (2017). Critical Assessment of Metagenome Interpretation-a benchmark of metagenomics software. Nat. Methods.

[20] Di Rienzi SC (2013). The human gut and groundwater harbor non-photosynthetic bacteria belonging to a new candidate phylum sibling to Cyanobacteria. Elife.

[21] Hawley ER (2014). Metagenomes from two microbial consortia associated with Santa Barbara seep oil. Mar. Genomics.

[22] Hawley ER (2014). Metagenomic analysis of microbial consortium from natural crude oil that seeps into the marine ecosystem offshore Southern California. Stand. Genom. Sci..

[23] Quast C (2013). The SILVA ribosomal RNA gene database project: improved data processing and web-based tools. Nucleic Acids Res..

[24] Butterfield CN (2016). Proteogenomic analyses indicate bacterial methylotrophy and archaeal heterotrophy are prevalent below the grass root zone. PeerJ.

[25] R Core Team. *R:**A Language and Environment for Statistical Computing* (R Foundation for Statistical Computing, 2015).

[26] Weston, S. & Calaway, R. doMC: Foreach Parallel Adaptor for ‘parallel’ (2015); https://cran.r-project.org/web/packages/doMC

[27] Dowle, M., Srinivasan, A., Short, T., Saporta, S. L. & Antonyan, E. data.table: Extension of Data.frame (2015); https://cran.r-project.org/web/packages/data.table

[28] Wickham H (2009). ggplot2: Elegant Graphics for Data Analysis.

[29] Hyatt D, Locascio PF, Hauser LJ, Uberbacher EC (2012). Gene and translation initiation site prediction in metagenomic sequences. Bioinformatics.

[30] Brown CT (2015). Unusual biology across a group comprising more than 15% of domain Bacteria. Nature.

[31] Edgar RC (2010). Search and clustering orders of magnitude faster than BLAST. Bioinformatics.

[32] Buchfink B, Xie C, Huson DH (2015). Fast and sensitive protein alignment using DIAMOND. Nat. Methods.

[33] Altschul SF, Gish W, Miller W, Myers EW, Lipman DJ (1990). Basic local alignment search tool. J. Mol. Biol..

[34] Singer E (2016). Next generation sequencing data of a defined microbial mock community. Sci. Data.

[35] Peng Y, Leung HCM, Yiu SM, Chin FYL (2012). IDBA-UD: a de novo assembler for single-cell and metagenomic sequencing data with highly uneven depth. Bioinformatics.

[36] Langmead B, Salzberg SL (2012). Fast gapped-read alignment with Bowtie 2. Nat. Methods.

[37] Ultsch, A. & Mörchen, F. ESOM-Maps: Tools for Clustering, Visualization, and Classification with Emergent SOM (2005); http://databionic-esom.sourceforge.net

[38] Wrighton KC (2012). Fermentation, hydrogen, and sulfur metabolism in multiple uncultivated bacterial phyla. Science.

[39] Kanehisa M, Goto S (2000). KEGG: Kyoto Encyclopedia of Genes and Genomes. Nucleic Acids Res..

[40] Suzek BE, Huang H, McGarvey P, Mazumder R, Wu CH (2007). UniRef: comprehensive and non-redundant UniProt reference clusters. Bioinformatics.

[41] UniProt Consortium. (2015). UniProt: a hub for protein information. Nucleic Acids Res..

[42] Edgar RC (2004). MUSCLE: a multiple sequence alignment method with reduced time and space complexity. BMC Bioinformatics.

[43] Stamatakis A (2014). RAxML version 8: a tool for phylogenetic analysis and post-analysis of large phylogenies. Bioinformatics.

[44] Miller MA, Pfeiffer W, Schwartz T (2010). Creating the CIPRES Science Gateway for inference of large phylogenetic trees. Gatew. Comput. Environ. Work. (GCE).

[45] Nawrocki, E. P. *Structural RNA Homology Search and Alignment using Covariance Models* All Theses and Dissertations (ETDs) (Washington University in Saint Louis, School of Medicine, 2009).

[46] Paradis E, Claude J, Strimmer K (2004). APE: analyses of phylogenetics and evolution in R language. Bioinformatics.

