# Supplementary material for: Recovery of genomes from metagenomes via a dereplication, aggregation and scoring strategy
Source: Nat Microbiol. 2018 May 28;3(7):836–43. doi: 10.1038/s41564-018-0171-1 (PMC6786971; doi:10.1038/s41564-018-0171-1)
Supplement: Supplementary file 1 — Supplementary Figures 1–9, Supplementary Figure 12, Supplementary Tables 3 and 4 [file 41564_2018_171_MOESM1_ESM.pdf]

nature microbiology

SUPPLEMENTARY INFORMATION

https://doi.org/10.1038/s41564-018-0171-1

In the format provided by the authors and unedited.

Recovery of genomes from metagenomes via a dereplication, aggregation and scoring strategy

Christian M. K. Sieber[1,2], Alexander J. Probst[2], Allison Sharrar[2], Brian C. Thomas[2], Matthias Hess[3], Susannah G. Tringe[1]* and Jillian F. Banfield[2]*

[1]Department of Energy, Joint Genome Institute, Walnut Creek, CA, USA. [2]Department of Earth and Planetary Science, University of California, Berkeley, CA, USA. [3]Department of Animal Science, University of California, Davis, CA, USA. *e-mail: sgtringe@lbl.gov; jbanfield@berkeley.edu

© 2018 Macmillan Publishers Limited, part of Springer Nature. All rights reserved.

Recovery of genomes from metagenomes via a dereplication, aggregation, and scoring strategy

Christian M. K. Sieber[1,2], Alexander J. Probst[2], Allison Sharrar[2], Brian C. Thomas[2], Matthias Hess[3], Susannah G. Tringe[1*], Jillian F. Banfield[2*]

[1]Department of Energy Joint Genome Institute, Walnut Creek, CA 94598, USA

[2]Department of Earth and Planetary Science, University of California, Berkeley, CA 94720, USA

[3]Department of Animal Science, University of California, Davis, CA, 95616, USA

*To whom correspondence should be addressed.

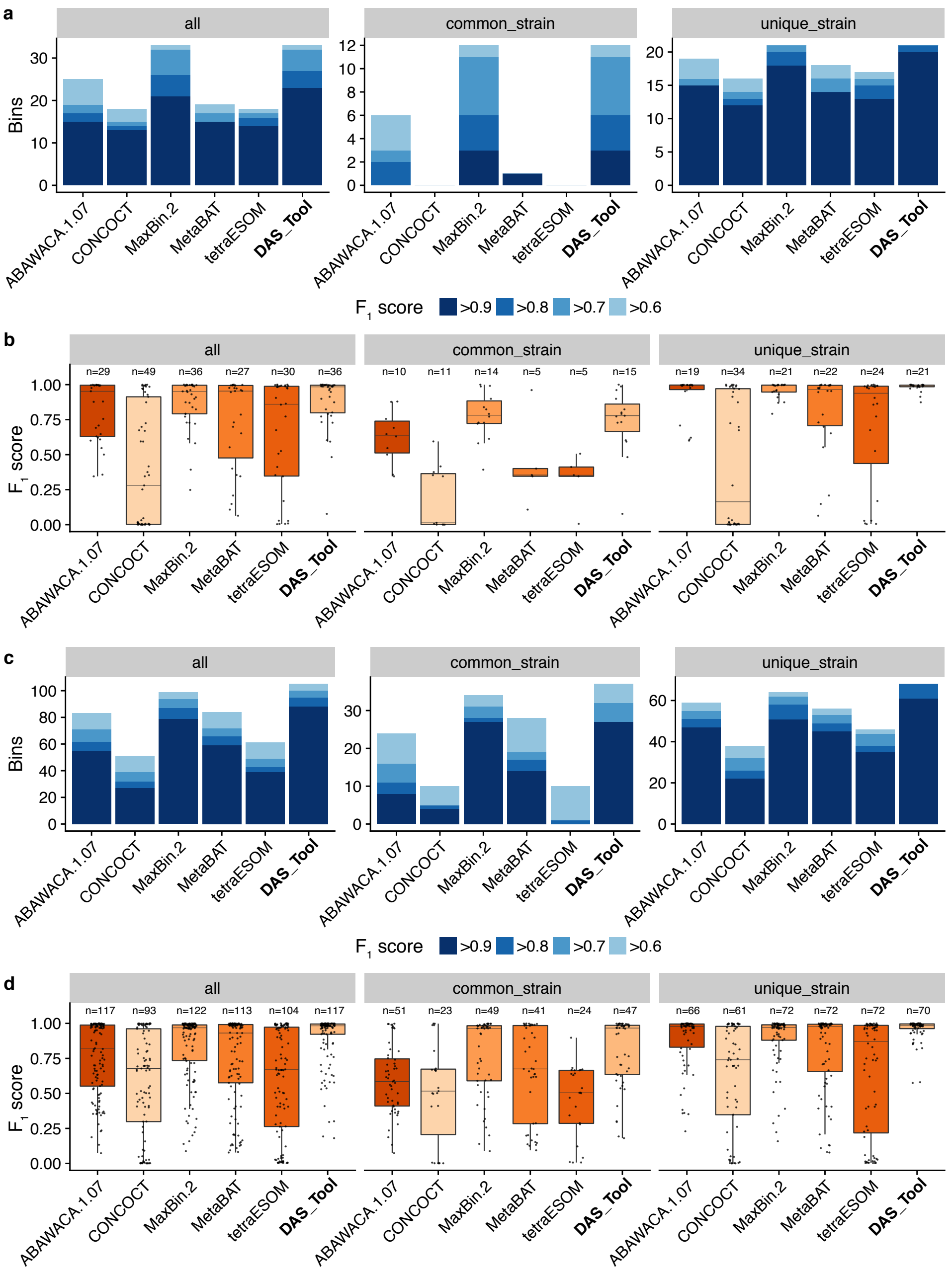

Supplementary Figure 1 Reconstructed genomes from two simulated microbial communities consisting of 131 genomes (a, b) and 377 genomes (c, d). a, c, Number of reconstructed genomes per method above a certain F_1 score threshold. The higher the F_1 score the more similar the reconstructed genome is to the reference. b, d, Distribution of F_1 scores of all reported bins (centre line: median; box limits: upper and lower quartiles; whiskers: 1.5x interquartile range). Individual values appear as dots. The precise n number in terms of reconstructed bins per method is given above each boxplot. Metrics are calculated for all reference genomes (all), genomes with strain variation (common_strain; ≤ 95% ANI to other reference genomes) and without strain variation (unique_strain; >95% ANI to other reference genomes). DAS_Tool represents the combination of the binning results of ABAWACA (hierarchical clustering using tetranucleotides, differential coverage, and marker genes), CONCOCT (Gaussian mixture models using tetranucleotides, and differential coverage), MaxBin 2 (expectation-maximization using tetranucleotides, differential coverage, and marker genes), MetaBAT (k-medoid clustering using tetranucleotide frequencies and differential coverage), tetraESOMs (emergent self-organizing maps based on tetranucleotide frequencies).

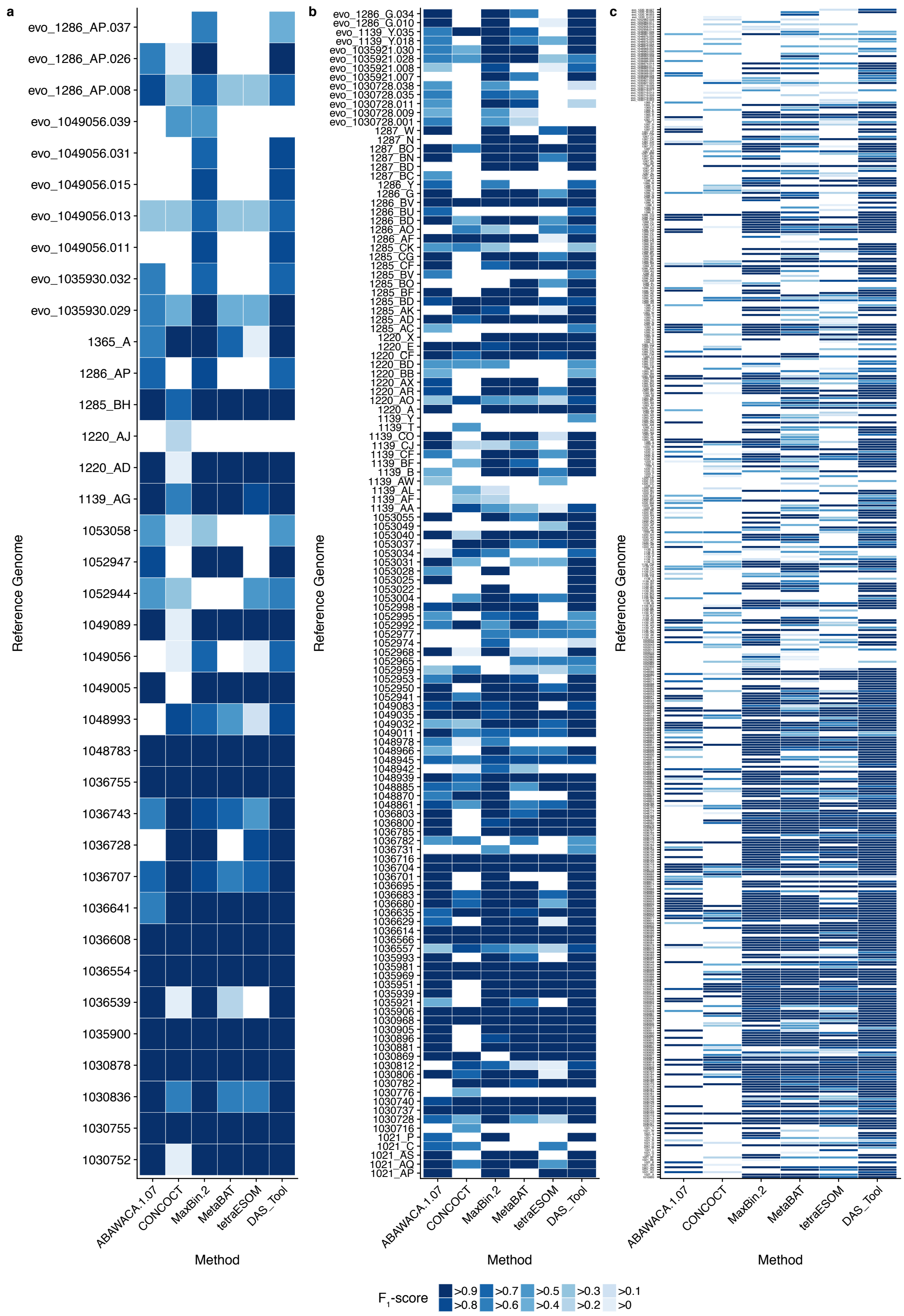

Supplementary Figure 2 Accuracy of reconstructed genomes per method based on F_1 score from simulated microbial communities of low (a), medium (b), and high complexity (c). F_1 scores were calculated using reference genomes. The higher the F_1 score the more similar the reconstructed genome is to the reference genome.

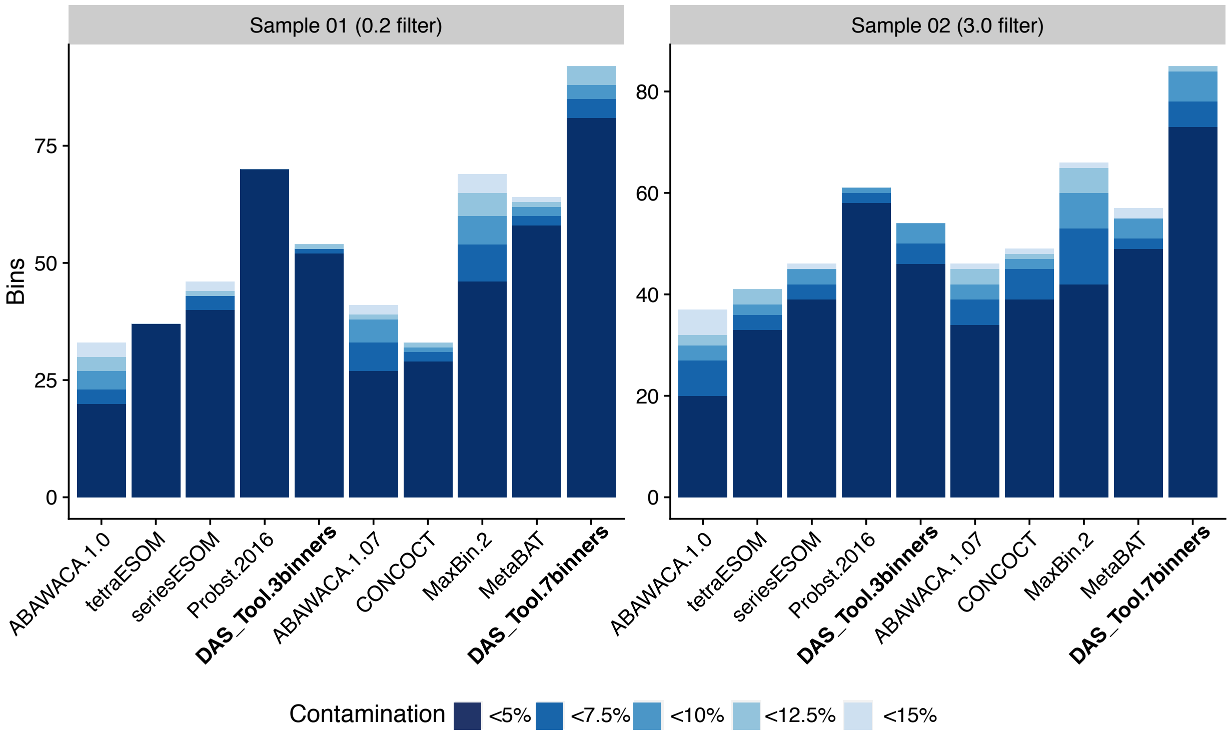

Supplementary Figure 3 Number of draft genomes with at least 70% completeness and less than 15% contamination for two real metagenomic assemblies from Crystal Geyser, a high CO_2 cold water geyser. Completeness and contamination was estimated using CheckM.

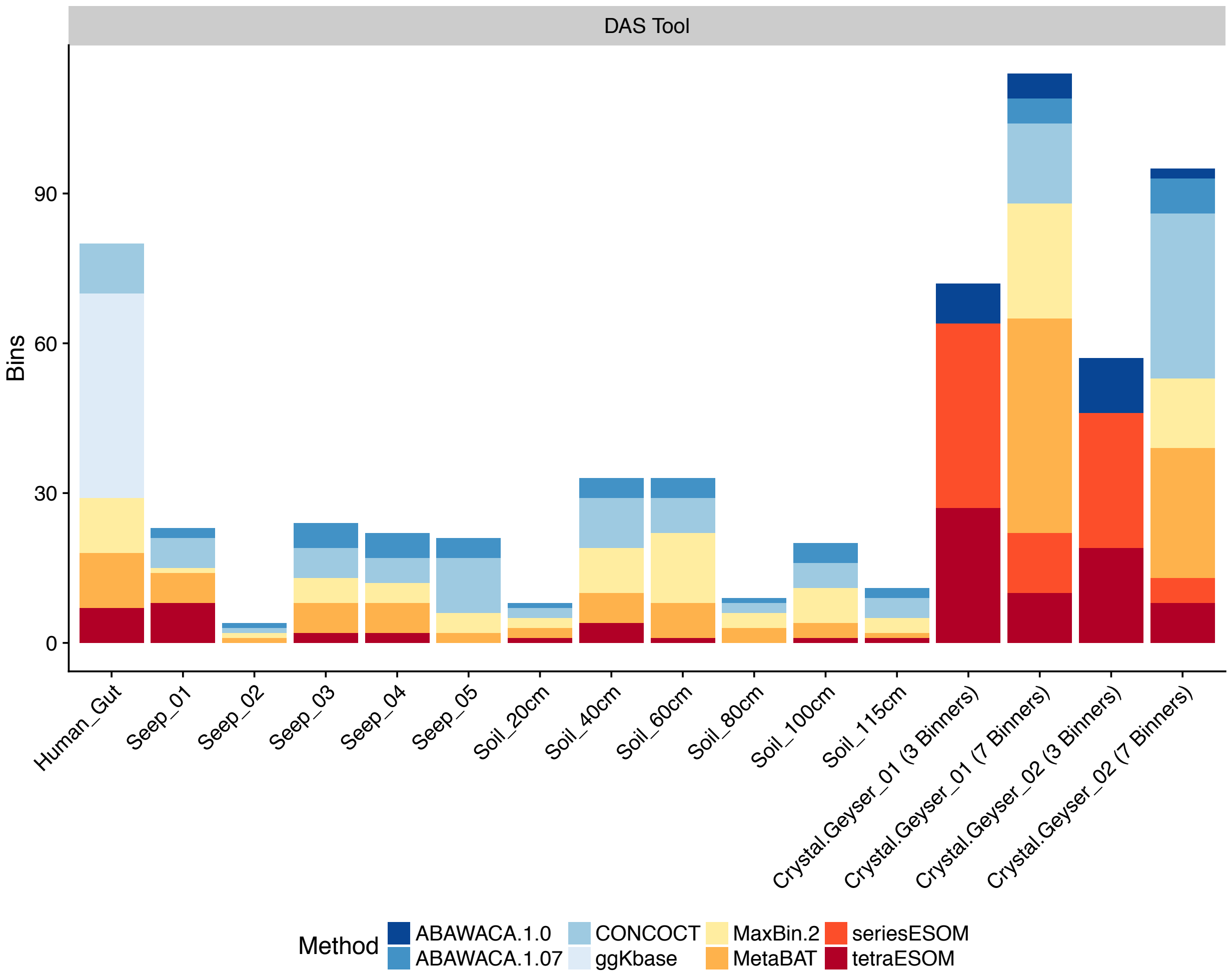

Supplementary Figure 4 Composition of reported bins (CheckM estimated Completeness > 60% and Contamination < 5%) by DAS Tool in terms of input binning method.

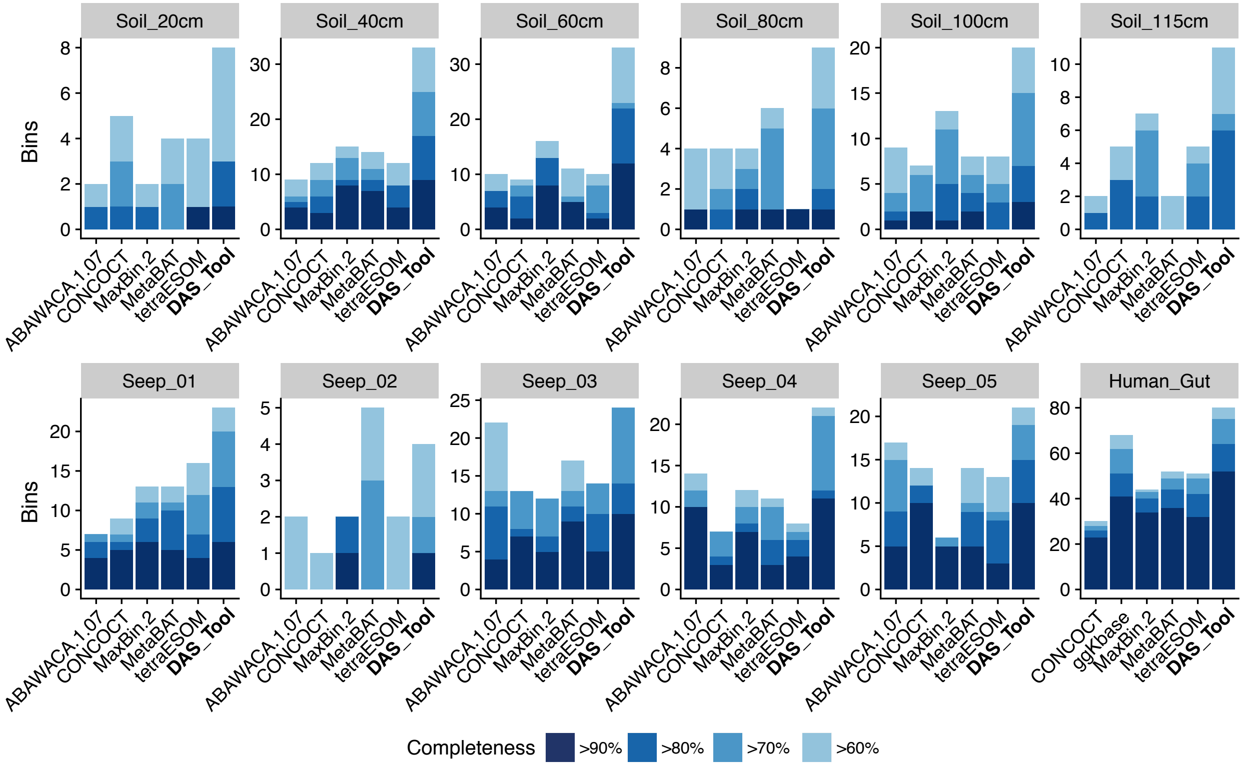

Supplementary Figure 5 Number of high quality genomes with low contamination (<5%) for twelve real metagenomic assemblies representing a range of complexity. Samples were collected from adult human gut, oil seeps and hillslope soil. Completeness and contamination was estimated using CheckM.

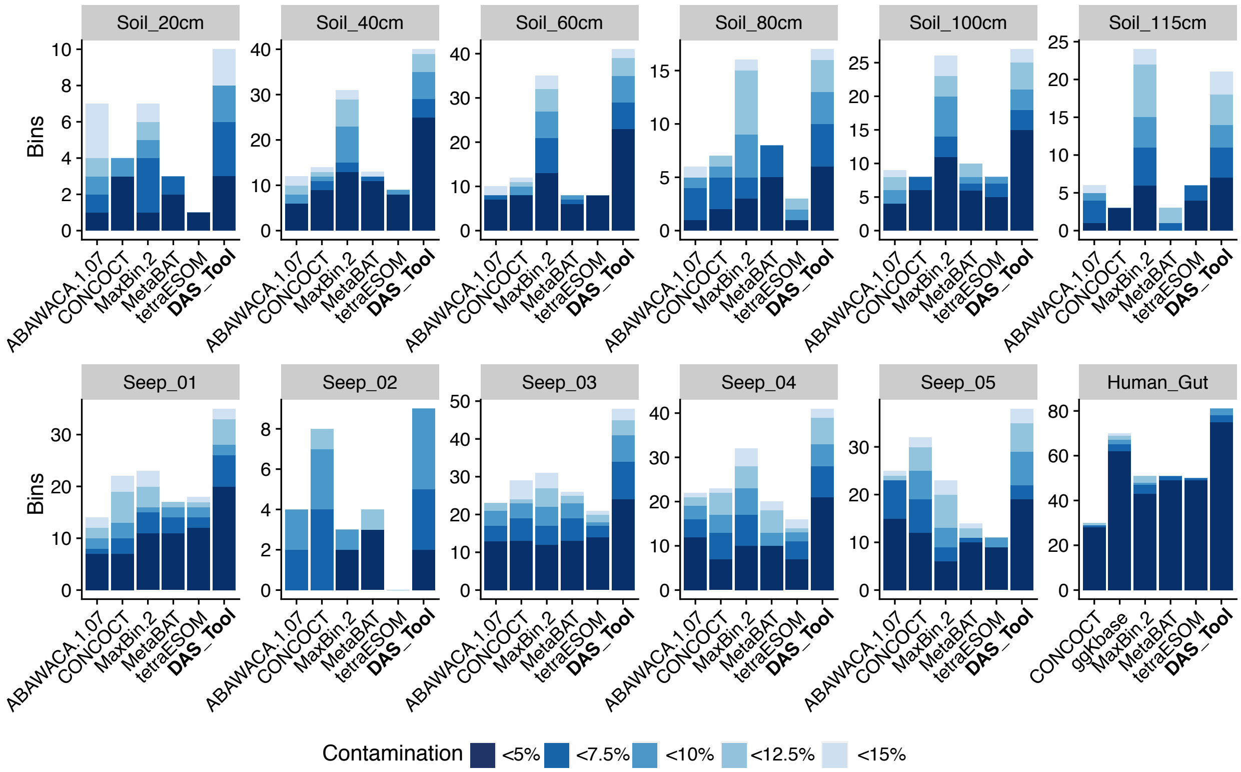

Supplementary Figure 6 Number of draft genomes with at least 70% completeness and less than 15% contamination for twelve real metagenomic assemblies representing a range of complexity. Samples were collected from adult human gut, oil seeps and hillslope soil. Completeness and contamination was estimated using CheckM.

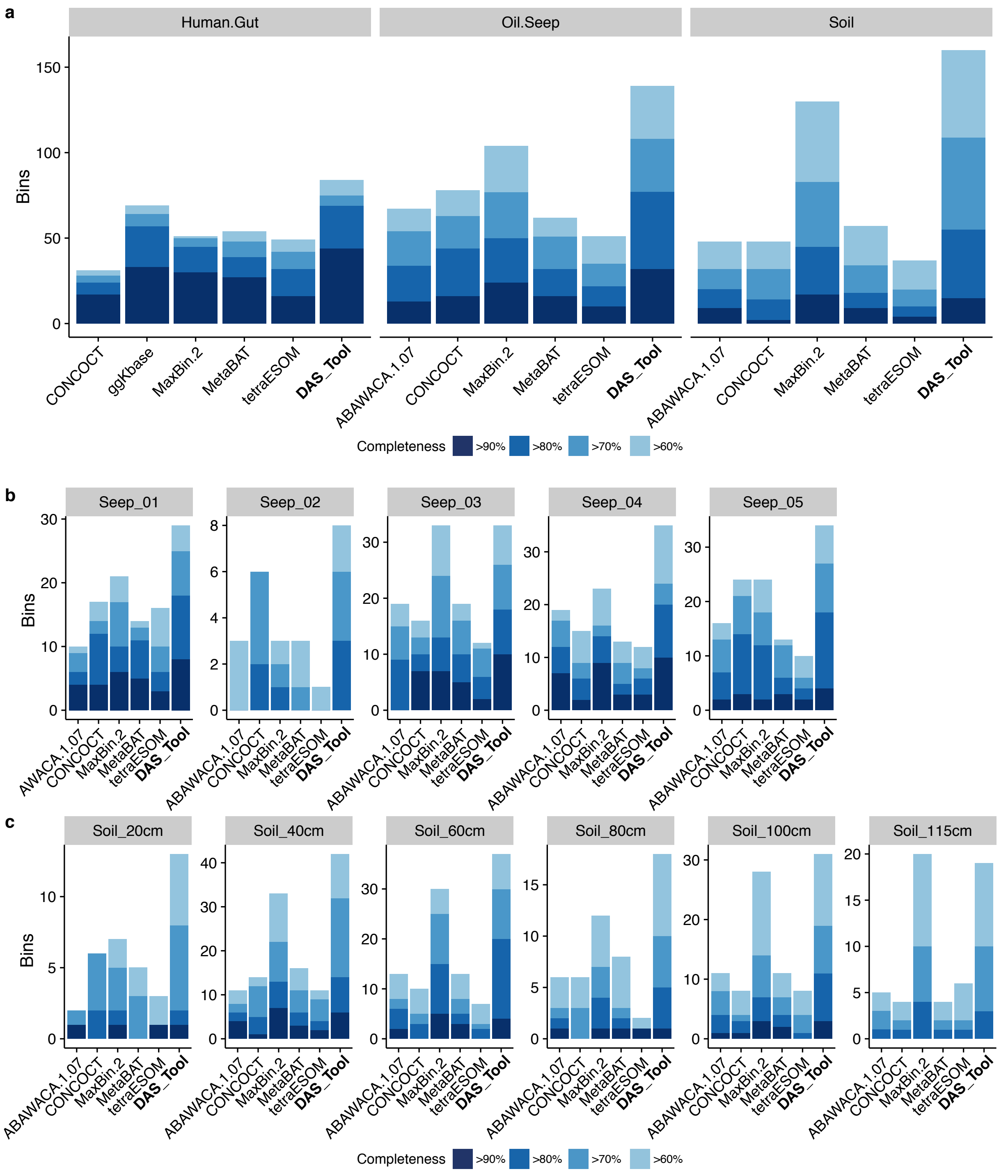

Supplementary Figure 7 Number of high quality genomes with low contamination (< 5%) from metagenomic assemblies of samples from three ecosystems representing a range of complexity. Completeness and contamination was estimated using BUSCO. Sum of reconstructed genomes per ecosystem (a). Samples were collected from adult human gut (1 sample), oil seeps (5 samples), hillslope soil and underlying weathered shale (6 samples). Number of reconstructed genomes per sample for oil seep (b) and soil (c).

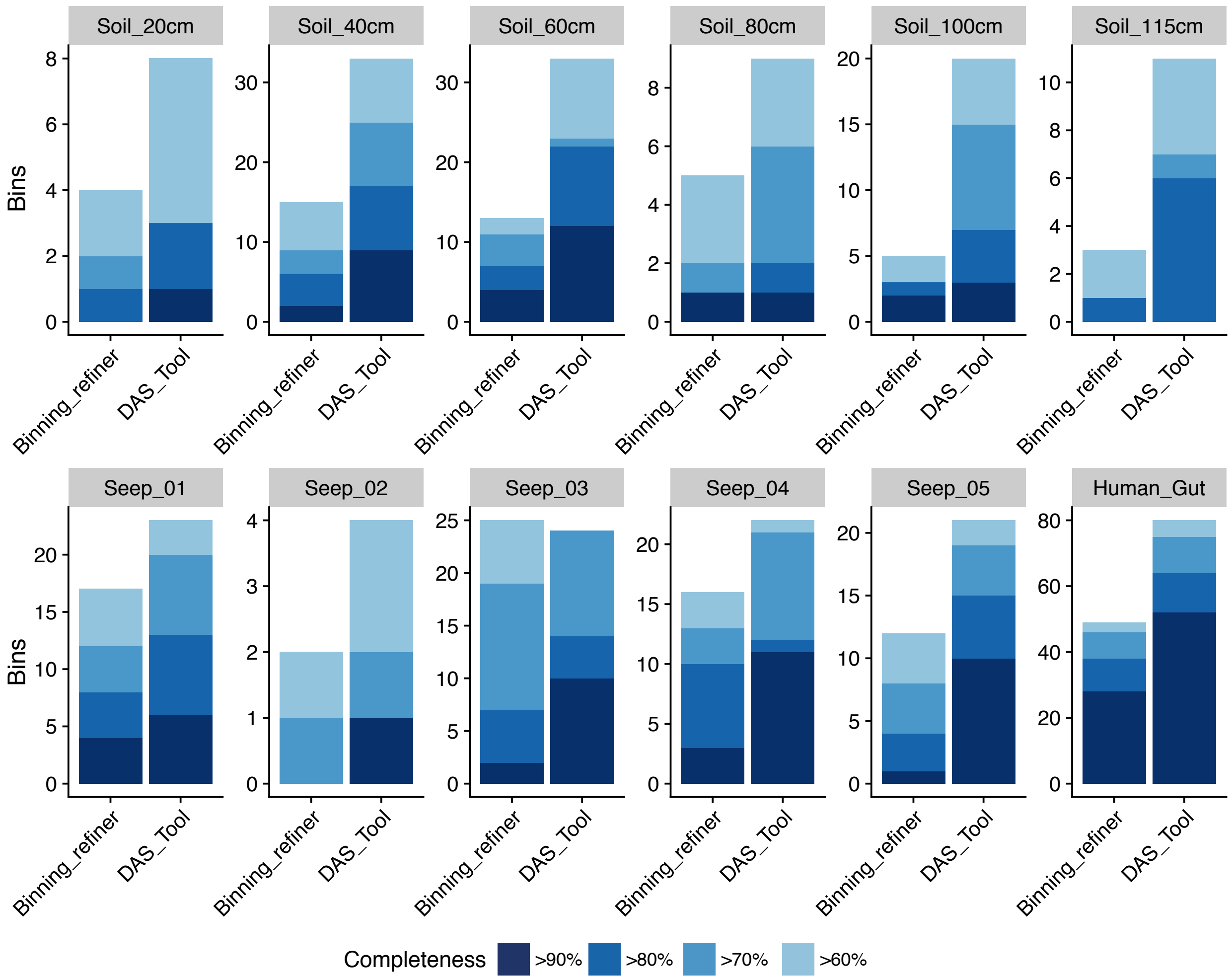

Supplementary Figure 8 Comparison of the performance of DAS Tool to Binning_refiner. Bins of five binners were combined using Binning_refiner and DAS Tool. Reconstructed number of high quality genomes with low contamination (<5%) from assemblies of human gut, natural oil seeps and soil was estimated using CheckM.

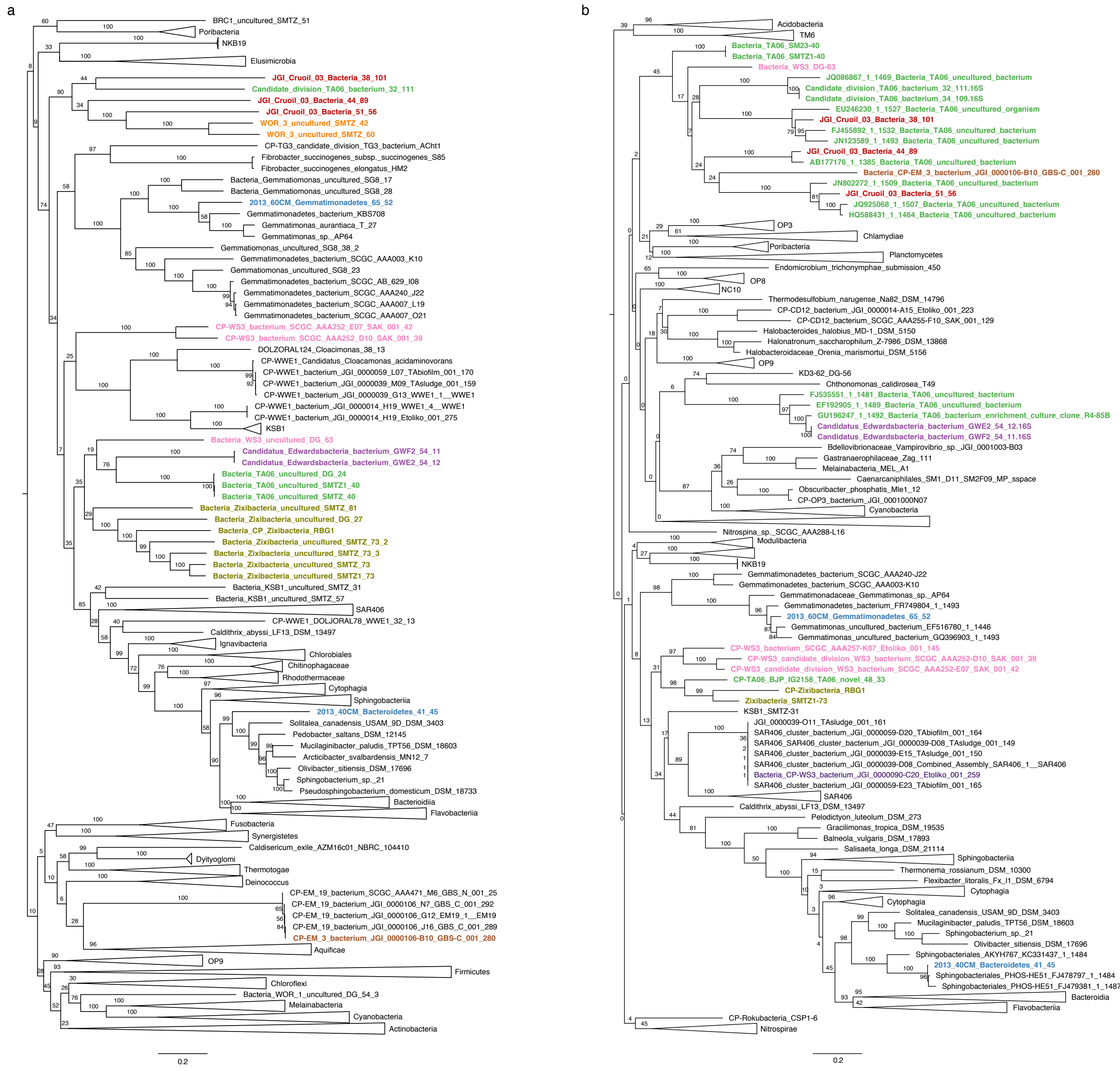

Supplementary Figure 9 Phylogenetic trees based on 16 concatenated ribosomal protein sequences (a) and based on 16S rRNA gene sequence showing reconstructed genomes from oil seeps (red) and soil metagenomes (blue). Reference genomes include TA06 (green), Edwardsbacteria (purple), WOR-3 (orange), WS-3 (pink), EM-3 (brown) and Zixibacteria (olive).

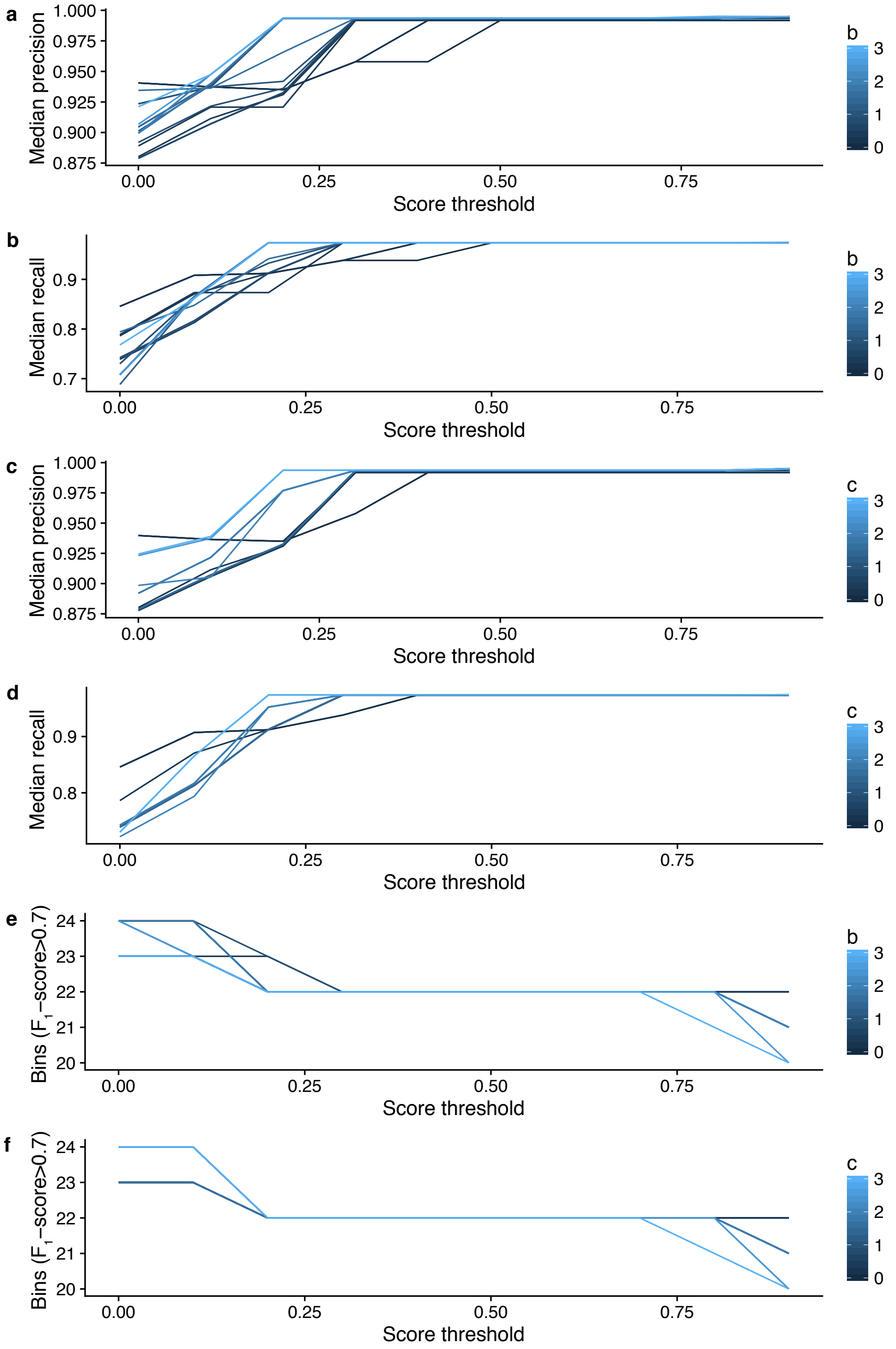

Supplementary Figure 12 Determination of parameters for DAS Tool. We applied DAS Tool with a range of values for the weighting parameters b, c, and the score threshold t on data from a synthetic microbial community. We calculated median precision and recall of reconstructed bins in dependence of variable values for b and t and a fixed value for c=0.5 (a,b) and variable values for c and t and a fixed value for b=0.5 (c,d). We also determined the number of accurately reconstructed bins (F_1 score > 0.7) for variable values for b and t and c=0.5 (e) and variable values for c and t and b=0.5 (f).

Supplementary Table 3 Number of reconstructed draft genomes (Completeness > 70%, Contamination < 5%) and estimated number of species per metagenomics assembly and ecosystem based on OTUs of ribosomal protein S3 (RPS3) sequences.

Environment	Sample	Draft genomes	RPS3-OTUs	% recovered
Oil Seeps	Seep_01	20	71	28.17%
	Seep_02	2	49	4.08%
	Seep_03	24	95	25.26%
	Seep_04	21	62	33.87%
	Seep_05	19	72	26.39%
Soil	Soil_20cm	3	101	2.97%
	Soil_40cm	25	191	13.09%
	Soil_60cm	23	139	16.55%
	Soil_80cm	6	68	8.82%
	Soil_100cm	15	222	6.76%
	Soil_115cm	7	186	3.76%
Human Gut	Human.gut	75	98	76.53%
Crystal Geyser	Geyser_01	81	225	36.00%
	Geyser_02	73	167	43.71%
Oil Seeps	5 Samples	86	349	24.64%
Soil	6 Samples	79	907	8.71%
Human Gut	1 Sample	75	98	76.53%
Crystal Geyser	2 Samples	154	392	39.29%

Supplementary Table 4 Genome quality estimates (CheckM) and 16S rRNA gene sequence similarities (SILVA) of reconstructed genomes from oil seeps.

Genome-Name	JGI_Cruoil_03_Bacteria_44_89	JGI_Cruoil_03_Bacteria_51_56	JGI_Cruoil_03_Bacteria_38_101
Completeness (CheckM)	95.6	93.55	89.64
Contamination (CheckM)	8.39	3.71	0.4
Strain.heterogeneity (CheckM)	87.5	88.89	0
sequence_score (SINA)	0.906799	0.955718	0.930279
bp_score (SINA)	107	108	105
cutoff_head (SINA)	0	0	0
cutoff_tail (SINA)	0	0	0
identity (SINA)	79.4243	87.448	78.7975
quality (SINA)	90	95	93
startpos (SINA)	1026	1026	1026
stoppos (SINA)	42621	42623	42621
ecolipos (SINA)	18	18	18
bps (SINA)	1414	1439	1450
gene_bps (SINA)	1414	1439	1450
turn (SINA)	none	none	none
lca_tax_embl (SINA)	Bacteria;	Bacteria;environmental samples;	Unclassified;
lca_tax_greengenes (SINA)	k__Bacteria;	k__Bacteria;p__AC1;c__SHA-114;	k__Bacteria;p__AC1;
lca_tax_ltp (SINA)	Bacteria;Unclassified Bacteria;Thermoanaerobaculum;	Unclassified;	Unclassified;
lca_tax_rdp (SINA)	Bacteria;	Bacteria;unclassified_Bacteria;	Bacteria;unclassified_Bacteria;
lca_tax_slv (SINA)	Bacteria;	Bacteria;AC1;	Bacteria;AC1;
