# Supplementary material for: Recovery of genomes from metagenomes via a dereplication, aggregation and scoring strategy
Source: Nat Microbiol. 2018 May 28;3(7):836–43. doi: 10.1038/s41564-018-0171-1 (PMC6786971; doi:10.1038/s41564-018-0171-1)
Supplement: Supplementary file 2 — Reporting Summary [file 41564_2018_171_MOESM2_ESM.pdf]

# nature research

Corresponding author(s): Jillian F. Banfield

☐ Initial submission ☒ Revised version ☐ Final submission

# Life Sciences Reporting Summary

Nature Research wishes to improve the reproducibility of the work that we publish. This form is intended for publication with all accepted life science papers and provides structure for consistency and transparency in reporting. Every life science submission will use this form; some list items might not apply to an individual manuscript, but all fields must be completed for clarity.

For further information on the points included in this form, see Reporting Life Sciences Research. For further information on Nature Research policies, including our data availability policy, see Authors & Referees and the Editorial Policy Checklist.

## Experimental design

1. Sample size

| | |
|---|---|
| Describe how sample size was determined. | We careful selected publicly available metagenomics datasets for testing. |

2. Data exclusions

| | |
|---|---|
| Describe any data exclusions. | No data was excluded from the selected metagenomics datasets. |

3. Replication

| | |
|---|---|
| Describe whether the experimental findings were reliably reproduced. | Our presented method in this paper outperforms existing methods in all 12 biological metagenomics samples and all three simulated communities. |

4. Randomization

| | |
|---|---|
| Describe how samples/organisms/participants were allocated into experimental groups. | No samples were allocated to experimental groups |

5. Blinding

| | |
|---|---|
| Describe whether the investigators were blinded to group allocation during data collection and/or analysis. | No blinding was appropriate |

Note: all studies involving animals and/or human research participants must disclose whether blinding and randomization were used.

6. Statistical parameters

For all figures and tables that use statistical methods, confirm that the following items are present in relevant figure legends (or in the Methods section if additional space is needed).

| n/a | Confirmed | |
|---|---|---|
| ☐ | ☒ | The exact sample size (*n*) for each experimental group/condition, given as a discrete number and unit of measurement (animals, litters, cultures, etc.) |
| ☒ | ☐ | A description of how samples were collected, noting whether measurements were taken from distinct samples or whether the same sample was measured repeatedly |
| ☒ | ☐ | A statement indicating how many times each experiment was replicated |
| ☒ | ☐ | The statistical test(s) used and whether they are one- or two-sided (note: only common tests should be described solely by name; more complex techniques should be described in the Methods section) |
| ☒ | ☐ | A description of any assumptions or corrections, such as an adjustment for multiple comparisons |
| ☒ | ☐ | The test results (e.g. *P* values) given as exact values whenever possible and with confidence intervals noted |
| ☒ | ☐ | A clear description of statistics including central tendency (e.g. median, mean) and variation (e.g. standard deviation, interquartile range) |
| ☒ | ☐ | Clearly defined error bars |

*See the web collection on statistics for biologists for further resources and guidance.*

## Software

Policy information about availability of computer code

7. Software

| | |
|---|---|
| Describe the software used to analyze the data in this | Our presented method, DAS Tool, is available on GitHub: https://github.com/cmks/ |

| | |
|---|---|
| study. | DAS_Tool. DAS Tool is an automated method that integrates a flexible number of binning algorithms to calculate an optimized, non-redundant set of genome bins from a single metagenome assembly. |

For manuscripts utilizing custom algorithms or software that are central to the paper but not yet described in the published literature, software must be made available to editors and reviewers upon request. We strongly encourage code deposition in a community repository (e.g. GitHub). *Nature Methods* guidance for providing algorithms and software for publication provides further information on this topic.

## ▸ Materials and reagents

Policy information about availability of materials

### 8. Materials availability

| | |
|---|---|
| Indicate whether there are restrictions on availability of unique materials or if these materials are only available for distribution by a for-profit company. | There are no restrictions on the availability of materials and data. |

### 9. Antibodies

| | |
|---|---|
| Describe the antibodies used and how they were validated for use in the system under study (i.e. assay and species). | No antibodies were used. |

### 10. Eukaryotic cell lines

| | |
|---|---|
| a. State the source of each eukaryotic cell line used. | No eukaryotic cell lines were used. |
| b. Describe the method of cell line authentication used. | No eukaryotic cell lines were used. |
| c. Report whether the cell lines were tested for mycoplasma contamination. | No eukaryotic cell lines were used. |
| d. If any of the cell lines used are listed in the database of commonly misidentified cell lines maintained by ICLAC, provide a scientific rationale for their use. | No eukaryotic cell lines were used. |

## ▸ Animals and human research participants

Policy information about studies involving animals; when reporting animal research, follow the ARRIVE guidelines

### 11. Description of research animals

| | |
|---|---|
| Provide details on animals and/or animal-derived materials used in the study. | No animal studies were conducted. |

Policy information about studies involving human research participants

### 12. Description of human research participants

| | |
|---|---|
| Describe the covariate-relevant population characteristics of the human research participants. | No studies involving human research participants were conducted. |
