# Supplementary material for: Recovery of genomes from metagenomes via a dereplication, aggregation and scoring strategy
Source: Nat Microbiol. 2018 May 28;3(7):836–43. doi: 10.1038/s41564-018-0171-1 (PMC6786971; doi:10.1038/s41564-018-0171-1)
Supplement: Supplementary file 8 — Source code of DAS Tool. For latest version please see https://github.com/cmks/DAS_Tool [file 41564_2018_171_MOESM8_ESM.gz › DAS_Tool/doc/DAS_Tool_documentation.pdf]

DAS Tool version 1.1 documentation

1. Usage

```
$ ./DAS_Tool -i methodA.scaffolds2bin,...,methodN.scaffolds2bin
             -l methodA,...,methodN -c contigs.fa -o myOutput
```

Option	Short	Mandatory	Description
--bins	-i	X	Comma separated list of tab separated scaffolds to bin tables.
--contigs	-c	X	Contigs in fasta format.
--outputbasename	-o	X	Basename of output files.
--labels	-l		Comma separated list of binning prediction names.
--search_engine			Engine used for single copy gene identification [blast/usearch] (default usearch)
--write_bin_evals			Write evaluation for each input bin set [0/1] (default 1).
--create_plots			Create binning performance plots [0/1] (default 1).
--write_bins			Export bins as fasta files [0/1] (default 0).
--proteins			Predicted proteins in prodigal fasta format (>scaffoldID_geneNo). Gene prediction step will be skipped if given.
--score_threshold			Score threshold until selection algorithm will keep selecting bins [0..1] (default 0.5).
--duplicate_penalty			Penalty for duplicate single copy genes per bin (weight b). Only change if you know what you're doing. [0..3] (default: 0.6)
--megabin_penalty			Penalty for megabins (weight c). Only change if you know what you're doing. [0..3] (default: 0.5)
--threads	-t		Number of cpus to use (default 1).

--version	-v	Print version number and exit.
--help	-h	Print help page and exit.

1.1 Input file format

- Bins [--bins, -i]: Tab separated files of scaffold-IDs and bin-IDs. Scaffold to bin file example:

```
Scaffold_1  bin.01
Scaffold_8  bin.01
Scaffold_42 bin.02
Scaffold_49 bin.03
```

- Contigs [--contigs, -c]: Assembled contigs in fasta format:

```
>Scaffold_1
ATCATCGTCCGCATCGACGAATTCGGCGAACGAGTACCCCTGACCATCTCCGATTA...
>Scaffold_2
GATCGTCACGCAGGCTATCGGAGCCTCGACCCGCAAGCTCTGCGCCTTGGAGCAGG...
```

- Proteins (optional) [--proteins]: Predicted proteins in prodigal fasta format. Header contains scaffold-ID and gene number:

```
>Scaffold_1_1
MPRKNKKLPRHLLVIRTSAMGDVAMLPHALRALKEAYPEVKVTVATKSLFHPFFEG...
>Scaffold_1_2
MANKIPRVPVREQDPKVRATNFEEVCYGYNVEEATLEASRCLNCKNPRCVAACPVN...
```

1.2 Output files

- Summary of output bins including quality and completeness estimates (DASTool_summary.txt).
- Scaffold to bin file of output bins (DASTool_scaffolds2bin.txt).
- Quality and completeness estimates of input bin sets, if `--write_bin_evals 1` is set ([method].eval).
- Plots showing the amount of high quality bins and score distribution of bins per method, if `--create_plots 1` is set (DASTool_hqBins.pdf, DASTool_scores.pdf).
- Bins in fasta format if `--write_bins 1` is set (DASTool_bins).

1.3 Examples: Running DAS Tool on sample data.

Example 1: Run DAS Tool on binning predictions of MetaBAT, MaxBin, CONCOCT and tetraESOMs. Output files will start with the prefix *DASToolRun1*:

```
$ ./DAS_Tool -i sample_data/sample.human.gut_concoct_scaffolds2bin.tsv,
                sample_data/sample.human.gut_maxbin2_scaffolds2bin.tsv,
                sample_data/sample.human.gut_metabat_scaffolds2bin.tsv,
                sample_data/sample.human.gut_tetraESOM_scaffolds2bin.tsv
```

```
                -l concoct,maxbin,metabat,tetraESOM
                -c sample_data/sample.human.gut_contigs.fa
                -o sample_output/DASToolRun1
```

Example 2: Run DAS Tool again with different parameters. Use the proteins predicted in Example 1 to skip the gene prediction step, disable writing of bin evaluations, set the number of threads to 2 and score threshold to 0.6. Output files will start with the prefix *DASToolRun2*:

```
$ ./DAS_Tool -i sample_data/sample.human.gut_concoct_scaffolds2bin.tsv,
                sample_data/sample.human.gut_maxbin2_scaffolds2bin.tsv,
                sample_data/sample.human.gut_metabat_scaffolds2bin.tsv,
                sample_data/sample.human.gut_tetraESOM_scaffolds2bin.tsv
             -l concoct,maxbin,metabat,tetraESOM
             -c sample_data/sample.human.gut_contigs.fa
             -o sample_output/DASToolRun2
             --proteins sample_output/DASToolRun1_proteins.faa
             --write_bin_evals 0
             --threads 2
             --score_threshold 0.6
```

2. Dependencies

DAS Tool runs on Unix based operating systems like Linux or macOS (>10.6). It depends on:

- R (>= 3.2.3): https://www.r-project.org
- R-packages: data.table (>= 1.9.6), doMC (>= 1.3.4), ggplot2 (>= 2.1.0)
- ruby (>= v2.3.1): https://www.ruby-lang.org
- Pullseq (>= 1.0.2): https://github.com/bcthomas/pullseq
- Prodigal (>= 2.6.3): https://github.com/hyattpd/Prodigal
- coreutils (only macOS/ OS X): https://www.gnu.org/software/coreutils
- One of the following search engines:
  - USEARCH (>= 8.1): http://www.drive5.com/usearch/download.html
  - DIAMOND (>= 0.8.24): https://github.com/bbuchfink/diamond
  - BLAST+ (>= 2.5.0): https://blast.ncbi.nlm.nih.gov/Blast.cgi

3. Installation

3.1 Quick Installation

Download and extract DASTool.zip archive:

```
$ unzip DAS_Tool.v1.0.zip
$ cd ./DAS_Tool.v1.0
```

Install R-packages:

```
$ R CMD INSTALL ./package/DASTool_1.0.0.tar.gz
```

Download and extract SCG database:

```
$ wget http://banfieldlab.berkeley.edu/~csieber/db.zip
$ unzip db.zip
```

Run DAS Tool:

```
$ ./DAS_Tool -h
```

3.2 Detailed Installation

3.2.1 R and Ruby

Make sure R and ruby are installed. The following commands should return the version information of R and ruby that is installed on your system:

```
$ R --version
R version 3.3.2 (2016-10-31) -- "Sincere Pumpkin Patch"
Copyright (C) 2016 The R Foundation for Statistical Computing
Platform: x86_64-pc-linux-gnu (64-bit)
```

Information about installing R can be found here: https://www.r-project.org

```
$ ruby --version
ruby 2.3.1p112 (2016-04-26) [x86_64-linux-gnu]
```

Information about installing ruby can be found here: https://www.ruby-lang.org/en/documentation/installation

3.2.2 Tools for gene prediction and a protein search engine

Get **prodigal** from https://github.com/hyattpd/Prodigal and make sure the executable is available in your PATH. Therefore, you can add one of the following lines to your ~/.bash_profile:

```
export PATH="$PATH:/path/to/prodigal_folder"

DASTOOL_PRODIGAL="/path/to/prodigal_folder"
```

If everything is set up correctly you should get the version information prodigal after entering **prodigal -v** in you terminal:

```
$ prodigal -v
Prodigal V2.6.3: February, 2016
```

Get **pullseq** from https://github.com/bcthomas/pullseq and make sure the executable it is available in your PATH. Therefore you can add one of the following lines to your ~/.bash_profile:

```
export PATH="$PATH:/path/to/pullseq_folder"
```

or

```
DASTOOL_PULLSEQ="/path/to/pullseq_folder"
```

If everything is set up correctly you should get the version information prodigal after entering **pullseq --version** in you terminal:

```
$ pullseq --version
pullseq - a bioinformatics tool for manipulating fasta and fastq files
Version: 1.0.2              Name lookup method: UTHASH
```

Get **USEARCH** from http://www.drive5.com/usearch and make sure the executable it is available in your PATH. Therefore you can add one of the following lines to your ~/.bash_profile:

```
export PATH="$PATH:/path/to/usearch_folder"
```

or

```
DASTOOL_USEARCH="/path/to/usearch_folder"
```

If everything is set up correctly you should get the version information USEARCH after entering **usearch --version** in you terminal:

```
$ usearch --version
usearch v9.0.2132_i86linux32
```

Instead of USEARCH you can alternatively install **DIAMOND** (https://github.com/bbuchfink/diamond) and/or **BLAST** (https://blast.ncbi.nlm.nih.gov/Blast.cgi) and add the program folders to you path:

```
export PATH="$PATH:/path/to/diamond_folder"
export PATH="$PATH:/path/to/blast_folder"
```

or

```
DASTOOL_DIAMOND="/path/to/diamond_folder"
DASTOOL_BLAST="/path/to/blast_folder"
```

If everything is set up correctly you should get the version information DIAMOND/BLAST after entering **diamond --version** / **blastn -version** in you terminal:

```
$ diamond --version
diamond version 0.8.25

$ blastp -version
blastp: 2.5.0+
```

3.2.3 Install DAS Tool and dependent R-packages

Download and extract DASTool.zip archive:

```
$ unzip DASTool.zip
$ cd ./DASTool
```

Download and extract SCG database into the DAS Tool installation folder:

```
$ wget http://banfieldlab.berkeley.edu/~csieber/db.zip
$ unzip db.zip
```

Run R and install dependent R-packages **doMC**, **data.table** and **ggplot2** and their dependencies:

```
$ R
> repo='http://cran.us.r-project.org' #select a repository
> install.packages('doMC', repos=repo, dependencies = T)
> install.packages('data.table', repos=repo, dependencies = T)
> install.packages('ggplot2', repos=repo, dependencies = T)
> q() #quit R-session
```

After installing all dependent R-packages, the DAS Tool R-functions can be installed in a bash terminal:

```
$ R CMD INSTALL ./package/DASTool_1.0.0.tar.gz
```

or in an R-session:

```
$ R
> install.packages('package/DASTool_1.0.0.tar.gz')
> q() #quit R-session
```

Make sure DAS_Tool is executable:

```
$ chmod +x ./DAS_Tool
```

Now you are ready to run DAS Tool:

```
$ ./DAS_Tool --version
DAS Tool version 1.0
```

4. Troubleshooting

4.1 Dependencies not found

Problem: All dependencies are installed and the environmental variables are set but DAS Tool still claims that specific depencendies are missing.

Solution: Make sure that the dependency executable names are correct. For example USEARCH has to be executable with the command

```
$ usearch
```

If your USEARCH binary is called differently (e.g. usearch9.0.2132_i86linux32) you can either rename it or add a symbolic link called usearch:

```
$ ln -s usearch9.0.2132_i86linux32 usearch
```

5. About

DAS Tool Copyright (c) 2017, The Regents of the University of California, through Lawrence Berkeley National Laboratory (subject to receipt of any required approvals from the U.S. Dept. of Energy). All rights reserved.

If you have questions about your rights to use or distribute this software, please contact Berkeley Lab's Innovation and Partnerships department at IPO@lbl.gov referring to "DAS Tool (2017-024)".

NOTICE. This software was developed under funding from the U.S. Department of Energy. As such, the U.S. Government has been granted for itself and others acting on its behalf a paid-up, nonexclusive, irrevocable, worldwide license in the Software to reproduce, prepare derivative works, and perform publicly and display publicly. The U.S. Government is granted for itself and others acting on its behalf a paid-up, nonexclusive, irrevocable, worldwide license in the Software to reproduce, prepare derivative works, distribute copies to the public, perform publicly and display publicly, and to permit others to do so.
