# Supplementary figures and images for: Recovery of genomes from metagenomes via a dereplication, aggregation and scoring strategy
Source: Nat Microbiol. 2018 May 28;3(7):836–43. doi: 10.1038/s41564-018-0171-1 (PMC6786971; doi:10.1038/s41564-018-0171-1)

Score distribution of bins

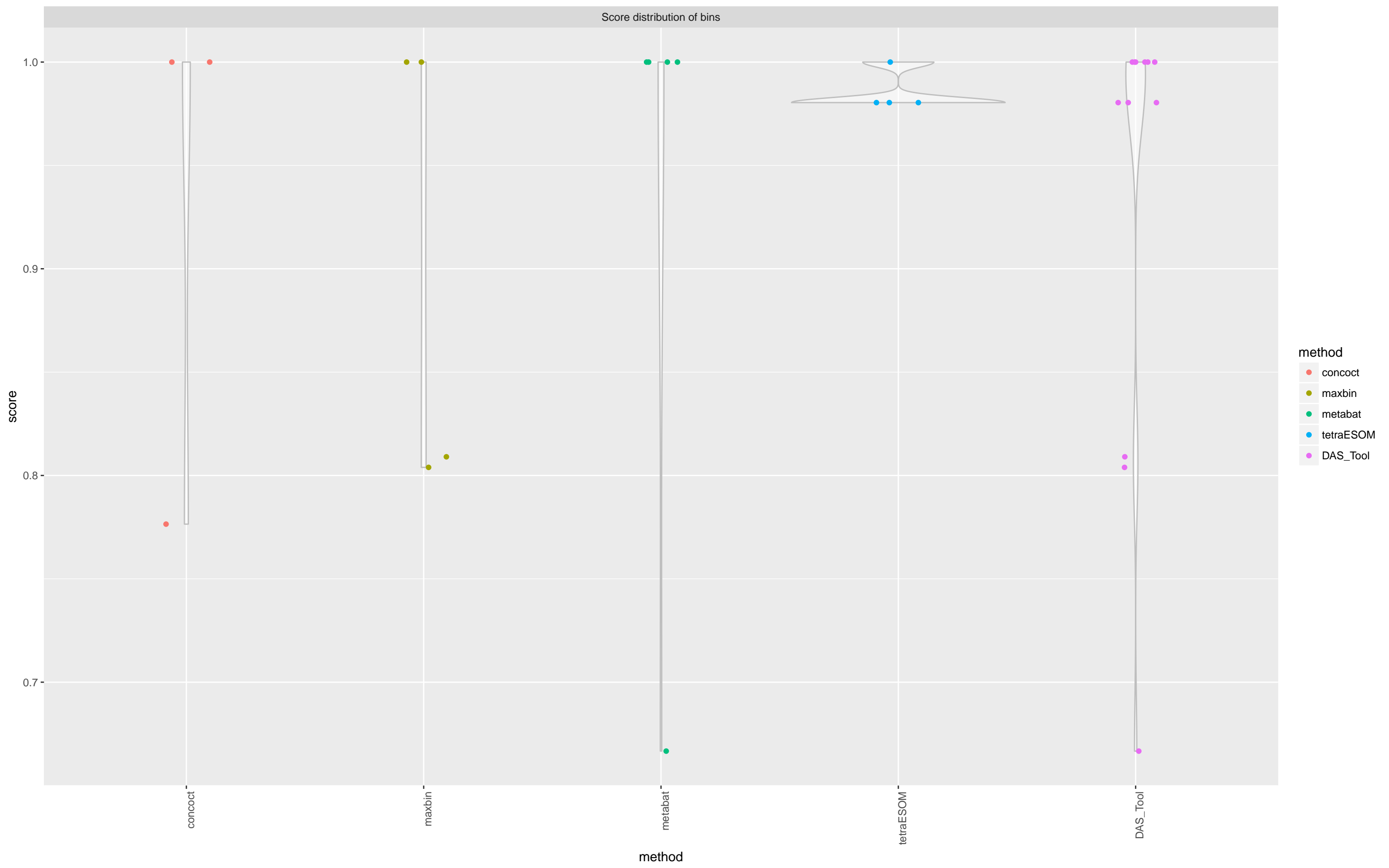

Supplement: Supplementary file 8 — Source code of DAS Tool. For latest version please see https://github.com/cmks/DAS_Tool [file 41564_2018_171_MOESM8_ESM.gz › DAS_Tool/sample_output/DASToolRun2_DASTool_scores.pdf]

Score distribution of bins

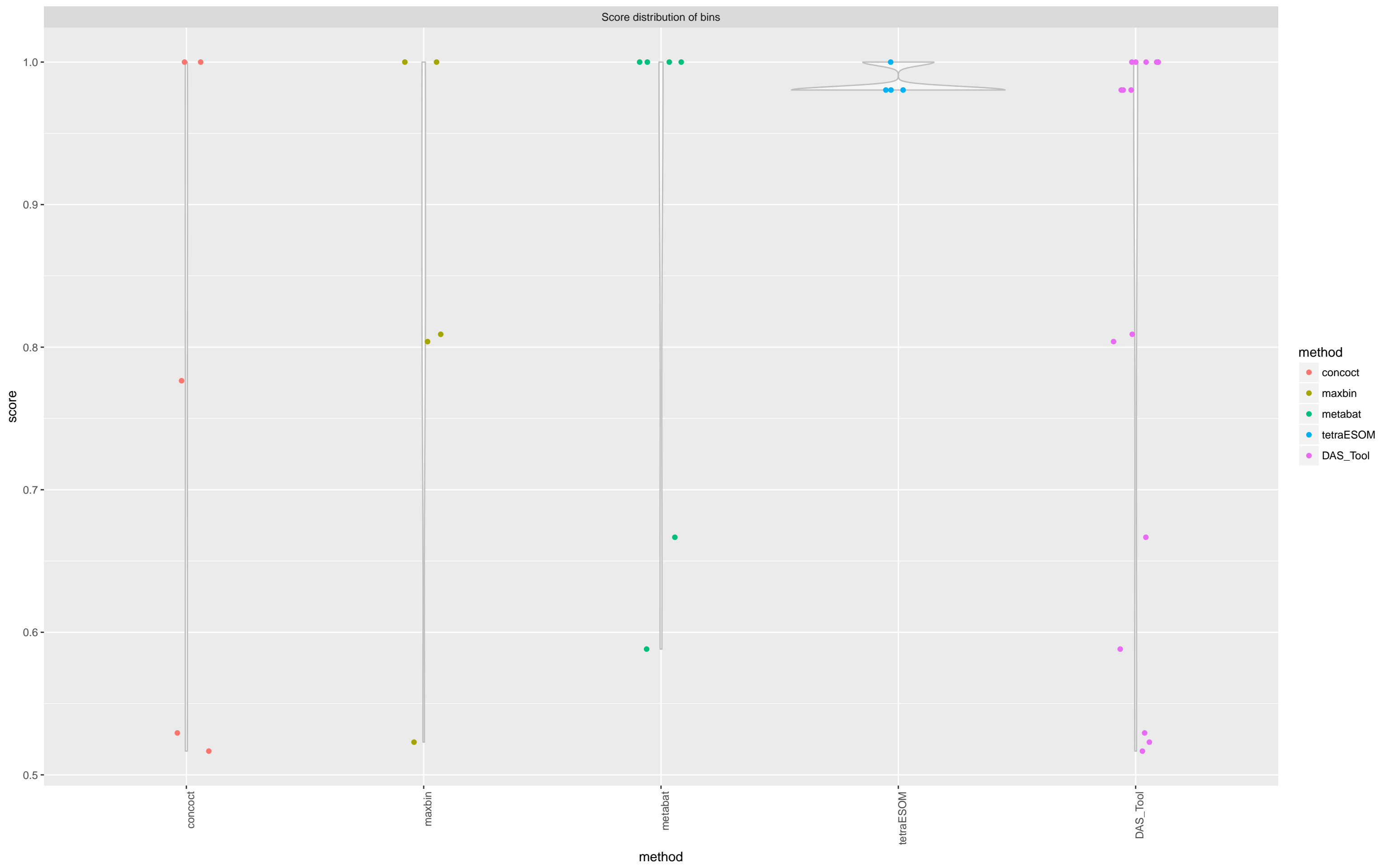

Supplement: Supplementary file 8 — Source code of DAS Tool. For latest version please see https://github.com/cmks/DAS_Tool [file 41564_2018_171_MOESM8_ESM.gz › DAS_Tool/sample_output/DASToolRun1_DASTool_scores.pdf]

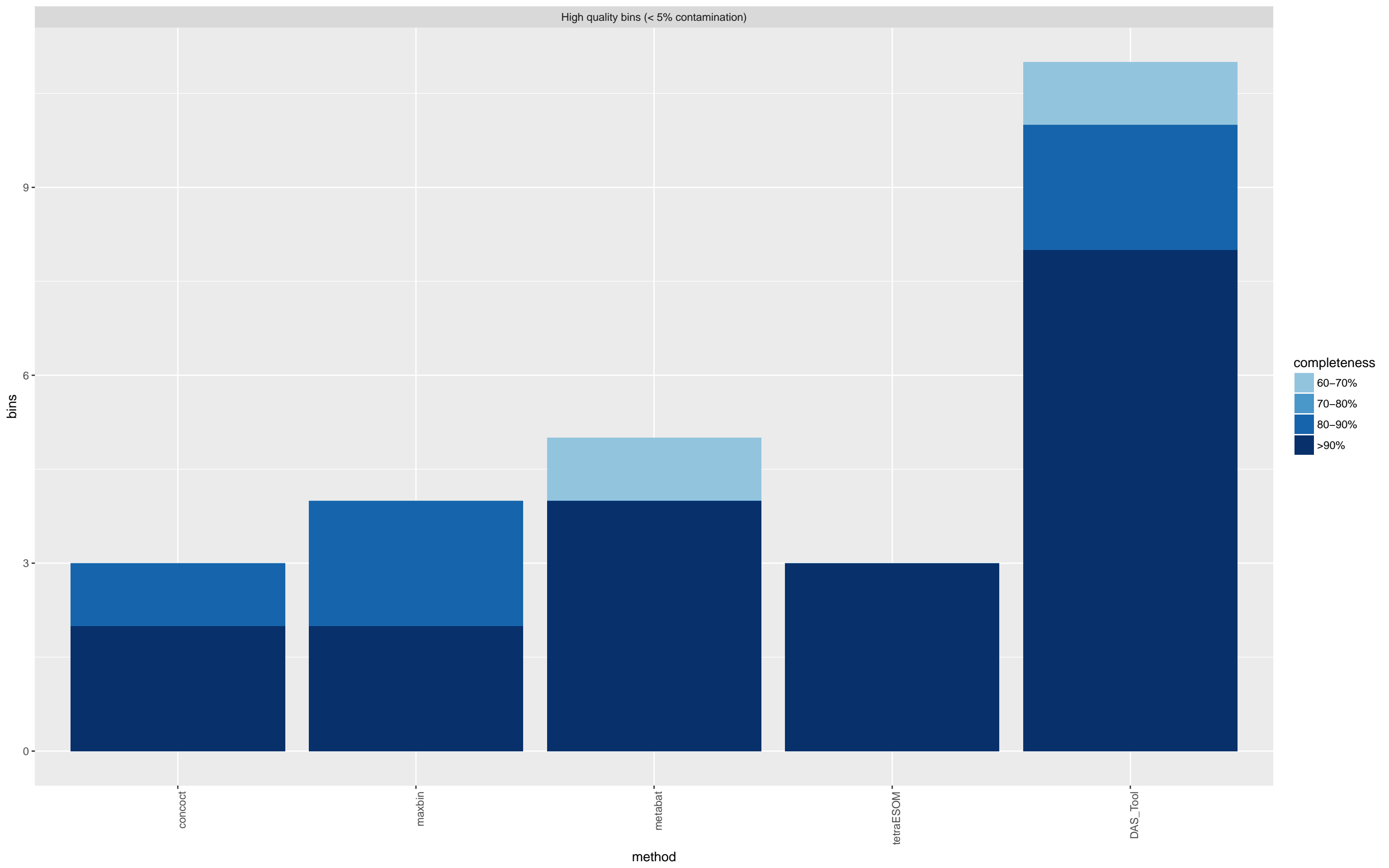

Supplement: Supplementary file 8 — Source code of DAS Tool. For latest version please see https://github.com/cmks/DAS_Tool [file 41564_2018_171_MOESM8_ESM.gz › DAS_Tool/sample_output/DASToolRun2_DASTool_hqBins.pdf]

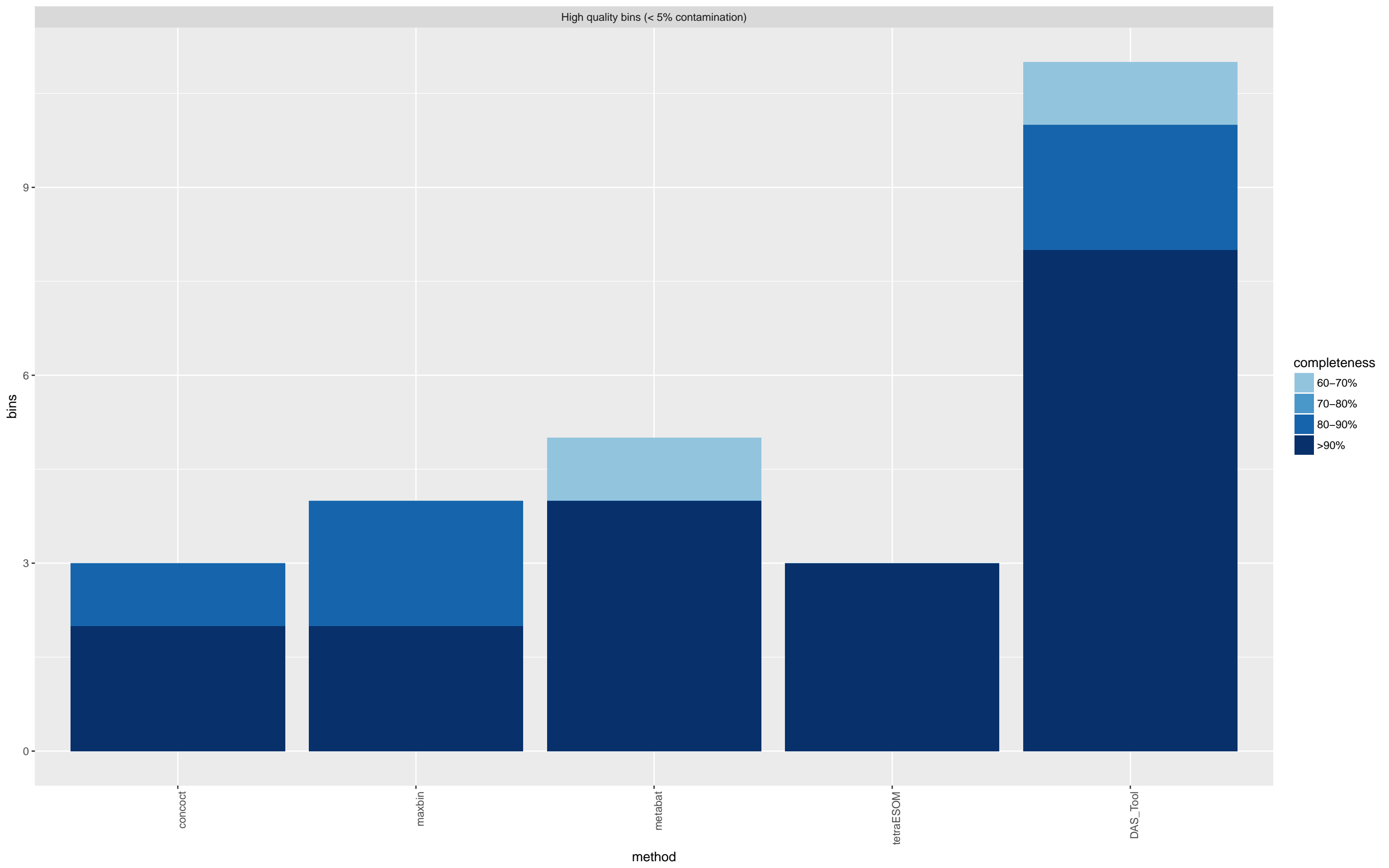

Supplement: Supplementary file 8 — Source code of DAS Tool. For latest version please see https://github.com/cmks/DAS_Tool [file 41564_2018_171_MOESM8_ESM.gz › DAS_Tool/sample_output/DASToolRun1_DASTool_hqBins.pdf]
