# Supplementary material for: Recovery of genomes from metagenomes via a dereplication, aggregation and scoring strategy
Source: Nat Microbiol. 2018 May 28;3(7):836–43. doi: 10.1038/s41564-018-0171-1 (PMC6786971; doi:10.1038/s41564-018-0171-1)

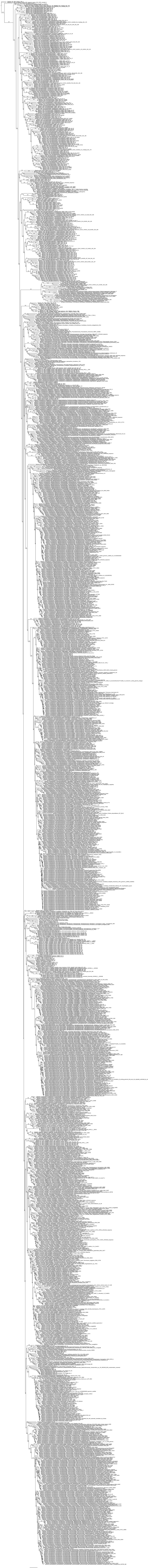

Supplement: Supplementary file 3 — Full-size phylogenetic trees based on 16 concatenated ribosomal protein sequences [file 41564_2018_171_MOESM3_ESM.pdf]
